# jViz.RNA 4.0—Visualizing pseudoknots and RNA editing employing compressed tree graphs

**DOI:** 10.1371/journal.pone.0210281

**Published:** 2019-05-06

**Authors:** Boris Shabash, Kay C. Wiese

**Affiliations:** School of Computing Science, Simon Fraser University, Burnaby, British Columbia, Canada; Erasmus Medical Center, NETHERLANDS

## Abstract

Previously, we have introduced an improved version of jViz.RNA which enabled faster and more stable RNA visualization by employing compressed tree graphs. However, the new RNA representation and visualization method required a sophisticated mechanism of pseudoknot visualization. In this work, we present our novel pseudoknot classification and implementation of pseudoknot visualization in the context of the new RNA graph model. We then compare our approach with other RNA visualization software, and demonstrate jViz.RNA 4.0’s benefits compared to other software. Additionally, we introduce interactive editing functionality into jViz.RNA and demonstrate its benefits in exploring and building RNA structures. The results presented highlight the new high degree of utility jViz.RNA 4.0 now offers. Users are now able to visualize pseudoknotted RNA, manipulate the resulting automatic layouts to suit their individual needs, and change both positioning and connectivity of the RNA molecules examined. Care was taken to limit overlap between structural elements, particularly in the case of pseudoknots to ensure an intuitive and informative layout of the final RNA structure.

**Availability**: The software is freely available at: https://jviz.cs.sfu.ca/.

## Introduction

### RNA structure

Ribo-nucleic Acid (RNA) is a polymer chain composed, mainly, of four bases called nucleotides. These are Adenine (A), Guanine (G), Cytosine (C), and Uracil (U). RNA most often appears as a single strand.

**Fig 1:**
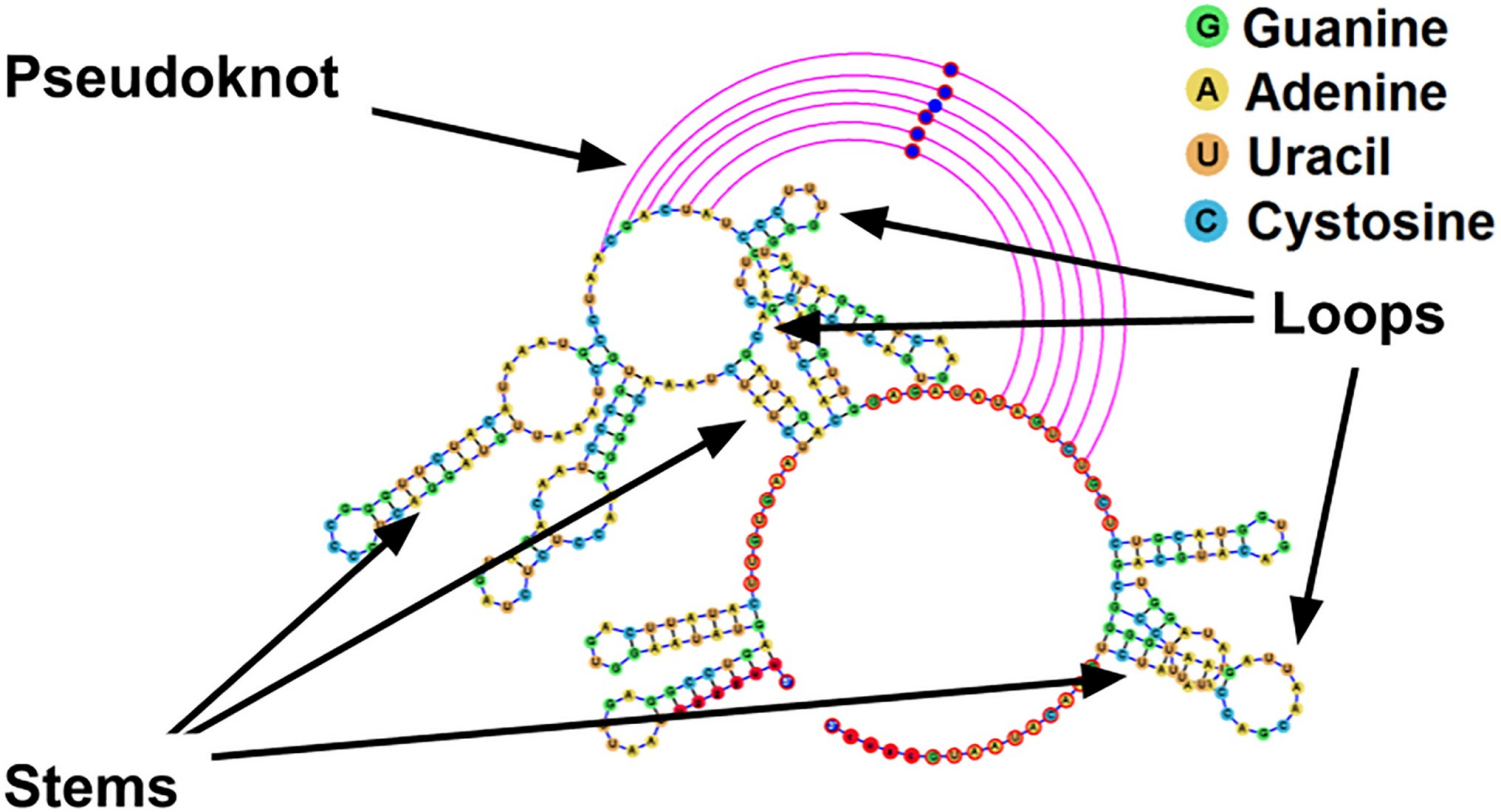
The secondary structure of the td group I intron pseudoknot (Accession ID: LC074723.1), with the different RNA structural motifs outlined. Sequence taken from http://pseudoviewer.inha.ac.kr/, examples tab, and image produced utilizing jViz.RNA 4.0.

The single RNA polymer can fold over itself to form base pairs between the complimentary nucleotides within the same strand. This process gives rise to the secondary structure of the RNA molecule, a sequence of paired nucleotides known as stems, and regions of unpaired bases known as loops. Furthermore, a tertiary structure known as a pseudoknot can be formed when a loop interacts with another loop or with a single stranded fragment which does not belong to any loop, bending the structure in 3D space. Fig 1 presents the mentioned RNA motifs.

RNA molecules are crucial agents in many biological processes. Some RNA molecules form structural motifs which allow viruses to manipulate host machinery [1–5], structures which produce antibiotic resistance in bacteria [6, 7], and even artificially synthesized RNA molecules designed to fight HIV [8].

The growing research into RNA function and shape created a need for software that can visualize RNA secondary structure to both explore potential experiments, and communicate the results of experiments conducted.

### RNA secondary structure visualization

Research into RNA and its secondary structure produced a variety of software tools, and a comprehensive review of these tools can be found in [9, 10]. Examples of tools that are still available include 4SALE [11, 12], Assemble2 [13, 14], RNA2DMap [15], R2R [16], RNApdbee 2.0 [17, 18], TRAVeLer [19], and R-Chie [20]. However, these RNA visualization software lack in one or more of the following categories:

- Installation and/or operation of the software requires use of the command terminal.
- The software produces static visualizations which cannot be modified after images and annotations have been prepared.
- Pseudoknot visualization is not supported, or is not rendered well within the image
- Editing of the RNA molecule requires modification of the input text files which do not lend themselves to a mental translation into secondary structure. Any user performing this editing has to put considerable effort into translating the results of their editing into visual representation while performing the edit operations.

Overall, only five notable software tools provide a dynamic structure which can be manipulated by the user, combined with support for pseudoknotted structures: PseudoViewer [21–25], jViz.RNA 2.0 [26–28], VARNA [29], RiboSketch [30], and Forna [31]. Of these tools, only RiboSketch [30] and Forna [31] allow for interactive editing of the RNA structures, while the other three software required the editing of the input files, which we will refer to as non-interactive editing.

**Interactive** editing allows users to edit the RNA structure model directly using the software without editing the input files, while **non-interactive** editing requires editing the input files and re-loading them into the software to see a change in the RNA structure (VARNA [29] allows for editing of the molecule using the software, but requires users to edit a string representation of the molecule model rather than the model itself. Hence it is still classified under **non-interactive** editing). The main advantage of interactive editing is the ease with which actions such as adding and removing base-pairs can be executed, while non-interactive editing requires users to translate the input file strings into a mental image of the RNA to introduce changes to the molecule. This inconveniences users in two ways: First, the mental translation from the text based input file to the secondary structure can be cumbersome, and second, the user must be familiar with the structure of input files.

**Fig 2:**
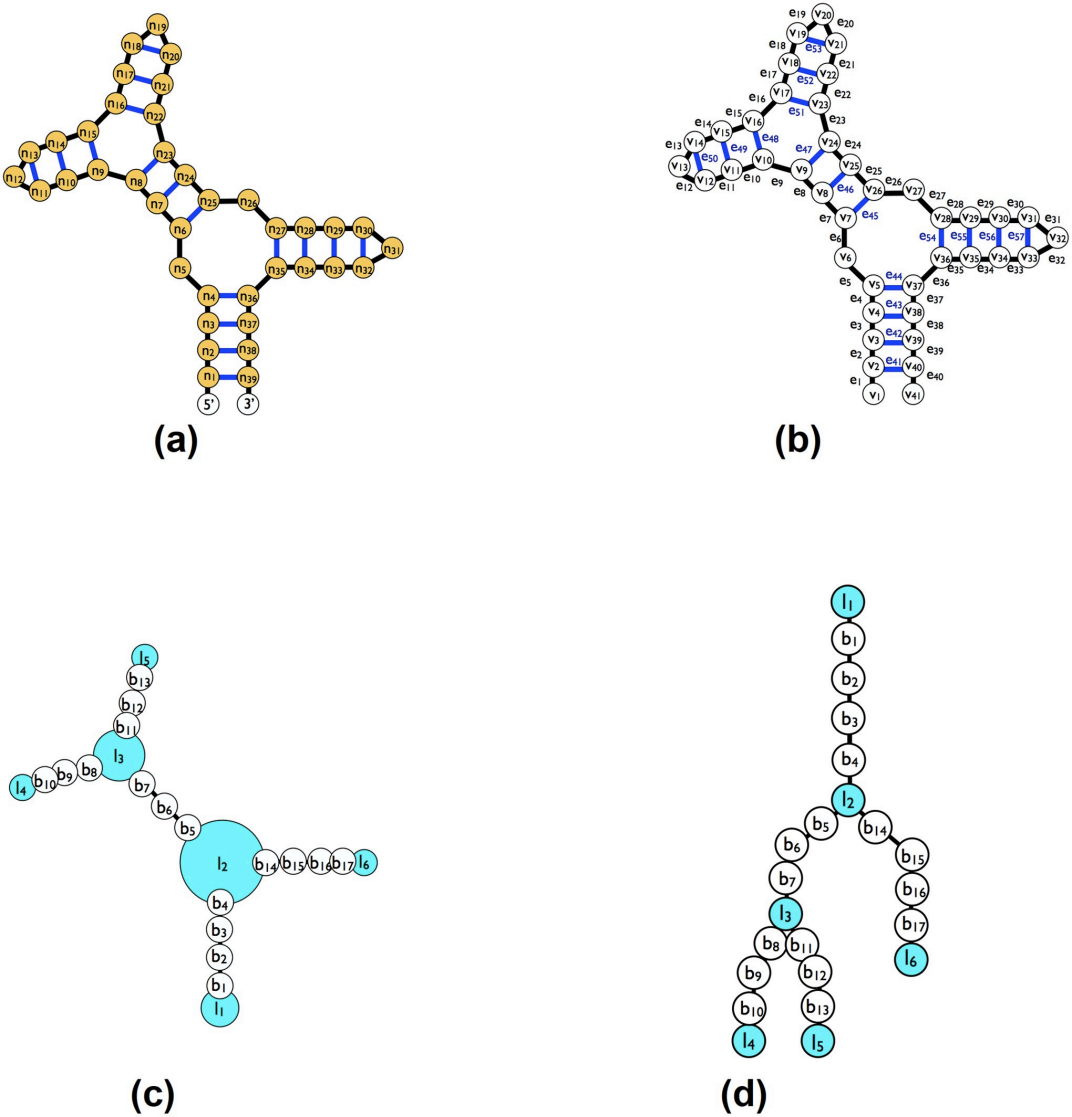
The compressed and detailed graph representations for a sample RNA molecule. (a) A sample RNA structure molecule. (b) The RNA molecule underlying representation used in RiboSketch [30] and Forna [31] (detailed graph). (c) The underlying representation of the RNA molecule in jViz.RNA 4.0 (compressed graph). (d) The representation also follows a tree structure where the first loop (which will always contain the 3’ and 5’ nucleotides) is the root, and the loops with only one stem attached to them always acting as leaves in the tree.

In previous work [32], we have described jViz.RNA 3.0, which employed a new underlying graph model to visualize the RNA molecule. Instead of translating the RNA molecule (Fig 2a) into a **detailed** graph where each nucleotide mapped to a node and each chemical base-pair between the nucleotides in the structure (hydrogen or covalent) mapped to an edge (Fig 2b), we introduced a **compressed** graph (Fig 2c) which mapped each loop or base-pair to a node, and connected those with edges, creating a tree like structure (Fig 2d). The resulting software tool was able to provide aesthetically better RNA visualizations, and respond to user interaction much faster, and with greater stability, even for large molecules. However, it faced two main challenges still: Formulating the new and improved compressed graph meant that removing and adding base-pairs between bases required a more sophisticated reconstruction of the graph (while for detailed graphs, like those used by Forna [31], it would require a simple removal or addition of an edge), and secondly, creating a responsive, adjustable, representation of pseudoknots which would avoid obstructive overlap with the main structure also requires a more sophisticated layout approach, and present a non-trivial challenge.

In this manuscript, we present two important extensions to jViz.RNA 3.0 [32]. Dynamic pseudoknotted structure visualization, and **interactive** RNA editing capacities which allows users to manipulate the connectivity of the RNA structure explored. The remainder of this paper is organized as follows: The following section demonstrates the results of the research described in this manuscript. The subsequent section discusses the significance and novel contributions of the work presented in this manuscript and the implication of this work. Finally, the succeeding section describes the approaches and methods employed to incorporate pseudoknot visualization and RNA editing into jViz.RNA 4.0, while the conclusion section provides concluding remarks. The features and capabilities of the aforementioned different software are summarized in Table 1.

**Table 1:** A comparison of the different RNA visualization software and their properties.

Software Tool	Secondary structure visualization	Dynamic RNA model	Pseudoknot visualization supported	Pseudoknots uniquely handled	Interactive editing available	Installation required	Compressed graph representation
4SALE [12]	✓					No	N/A
Assemble2 [14]	✓		✓		✓	No	N/A
RNA2DMap [15]	✓					No	N/A
R2R [16]	✓		✓	✓		Yes	N/A
RNApdbee [18]	✓		✓	✓		No	N/A
TRAVeler [19]	✓		✓			No	N/A
R-Chie [20]			✓	✓		No	N/A
PseudoViewer 3 [23]	✓	✓	✓	✓		No	✓
VARNA [29]	✓	✓	✓			No	✓
RiboSketch [30]	✓	✓	✓		✓	No	
Forna [31]	✓	✓	✓		✓	No	
jViz.RNA 2.0 [28]	✓	✓	✓			No	
jViz.RNA 3.0 [32]	✓	✓				No	✓
jViz.RNA 4.0	✓	✓	✓	✓	✓	No	✓

## Results

### Pseudoknot visualization

**Fig 3:**
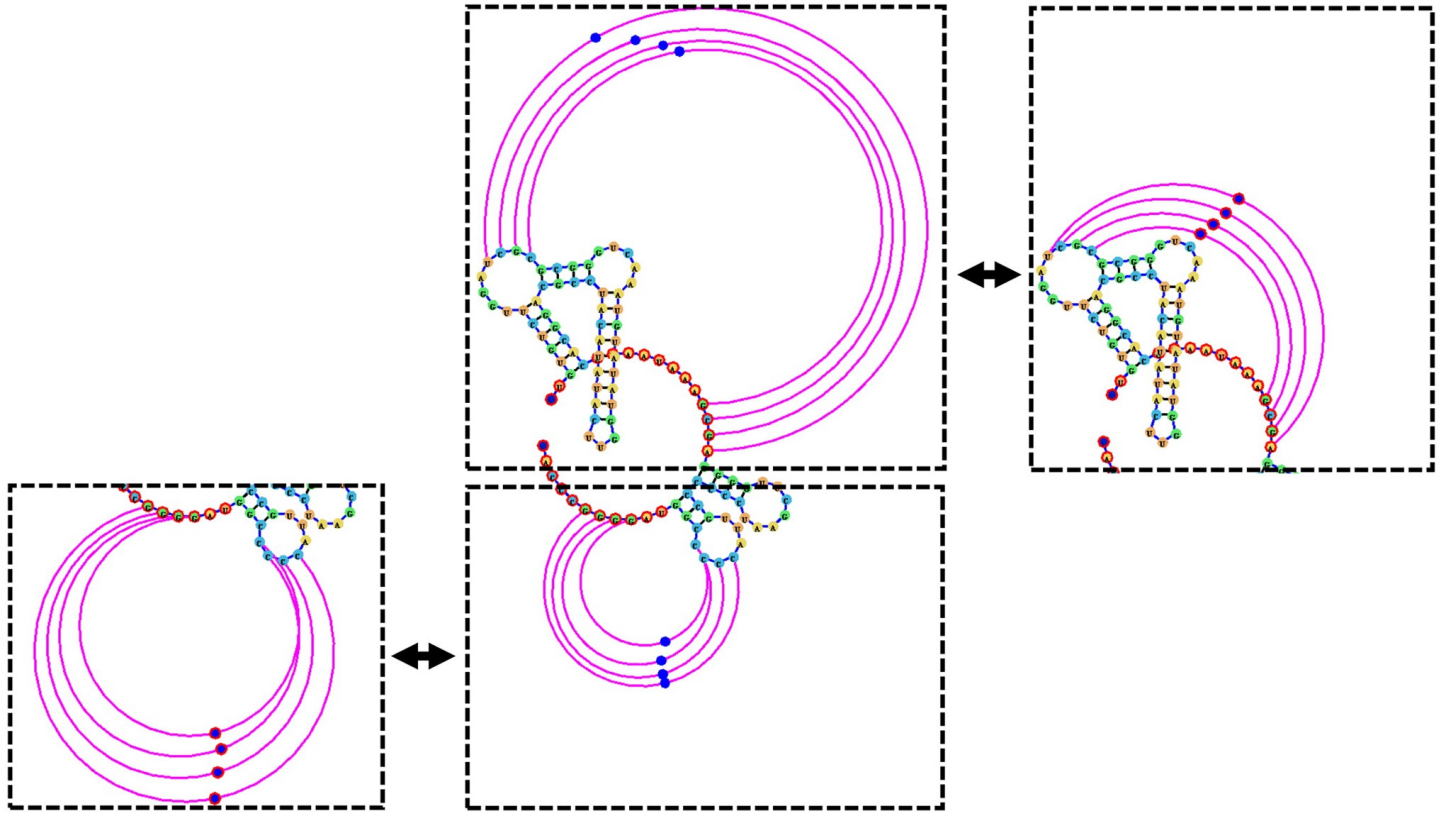
After the automatic layout of the helper nodes (blue spheres) has been computed (center image), users can drag the helper nodes to manipulate the pseudoknotted base-pairs to either reduce space (right image), or expand a pseudoknot to draw attention or isolate its base-pairs (left image).

**Fig 4:**
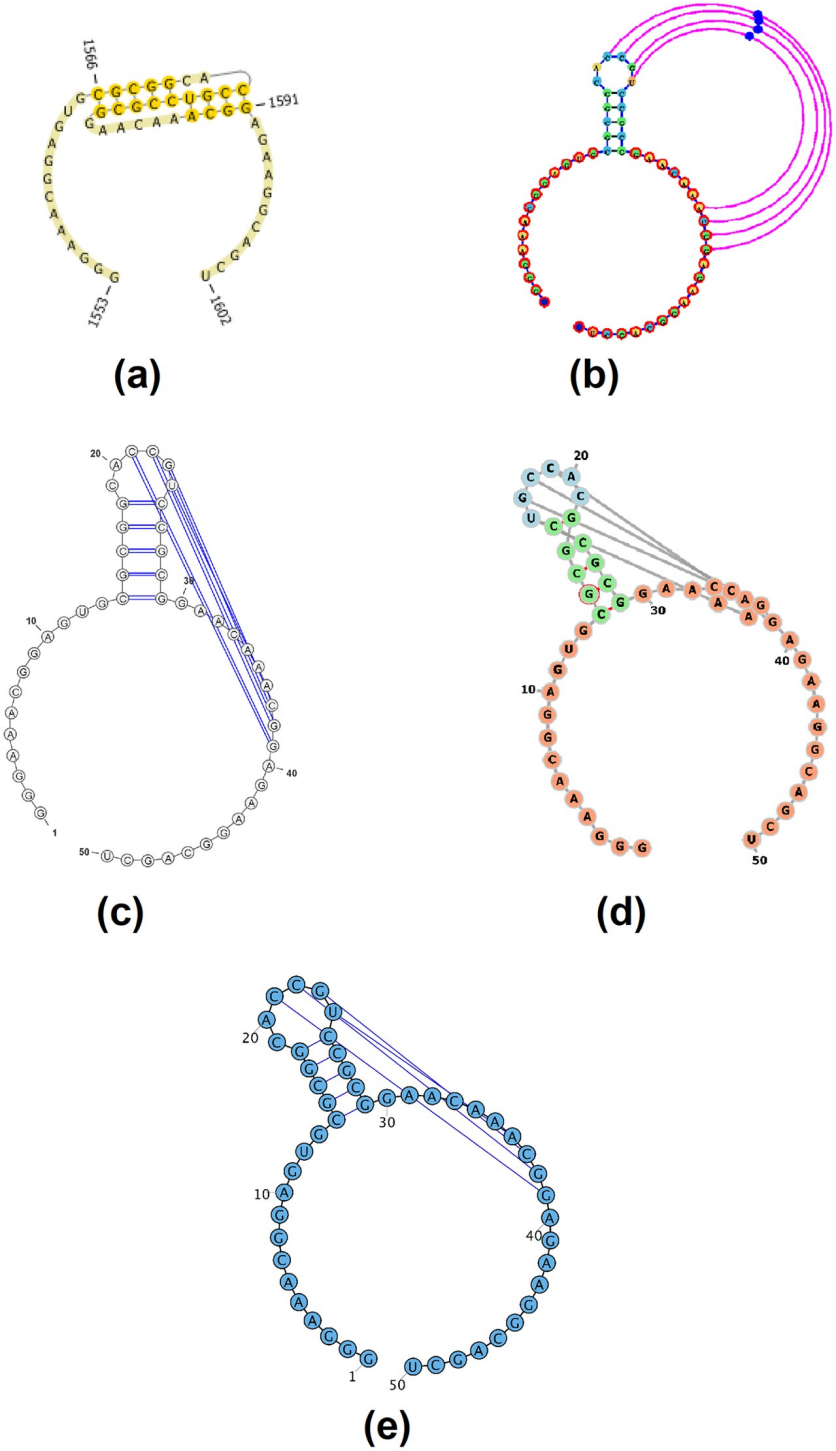
The resulting visualization of the Beet Western-Yellow Virus pseudoknot, labeled [AB] by jViz.RNA 4.0 (EMBL number X13063) [33–35] responsible for viral frameshifting utilizing: (a) PseudoViewer 3 [23], (b) jViz.RNA 4.0, (c) VARNA [29], (d) Forna [31], and (e) RiboSketch [30].

**Fig 5:**
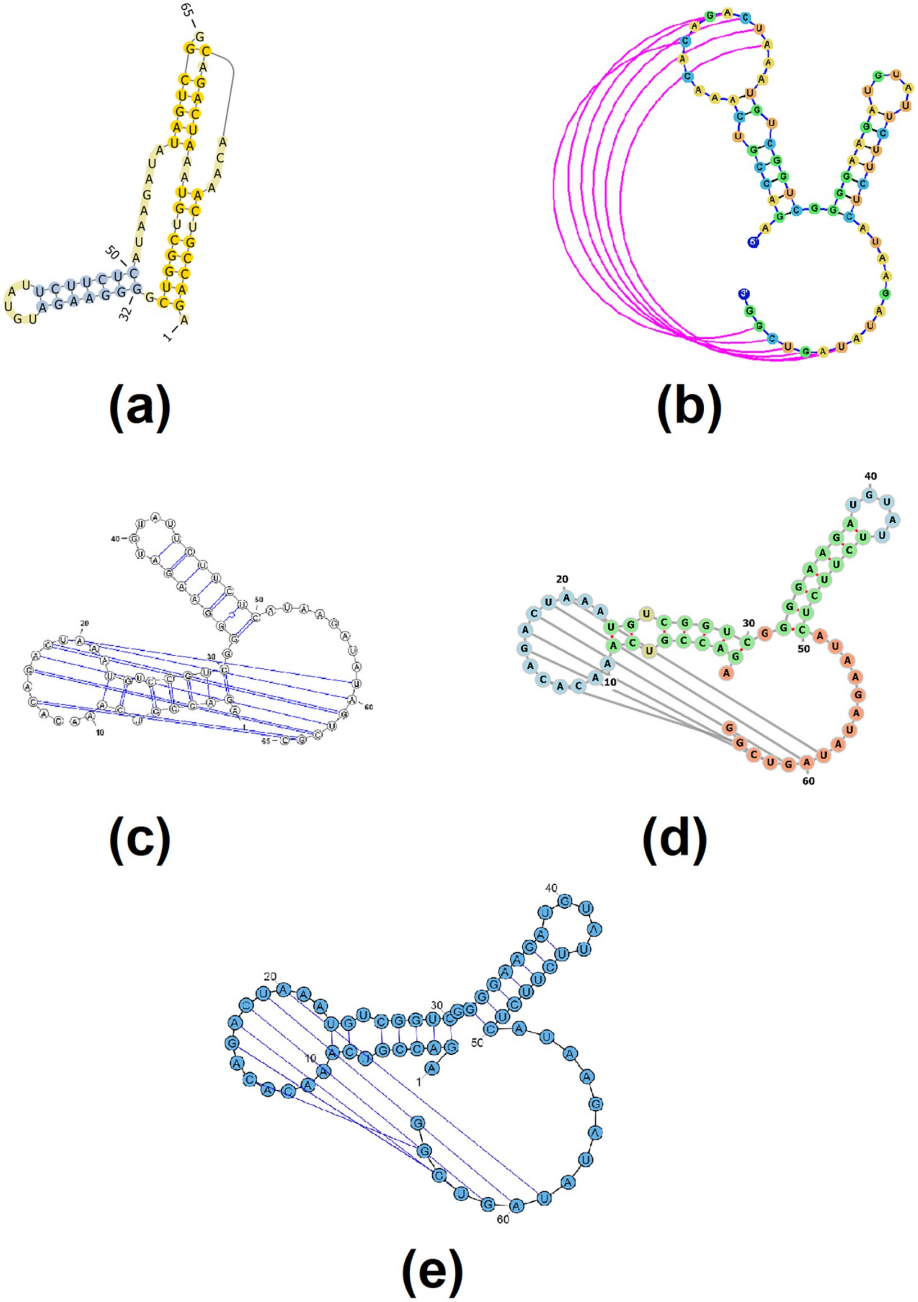
The resulting visualization of the *T. thermophila* pseudoknot, labeled [AB] by jViz.RNA 4.0 (EMBL number V01416) [36–38] ribozyme utilizing: (a) PseudoViewer 3 [23], (b) jViz.RNA 4.0, (c) VARNA [29], (d) Forna [31], and (e) RiboSketch [30].

**Fig 6:**
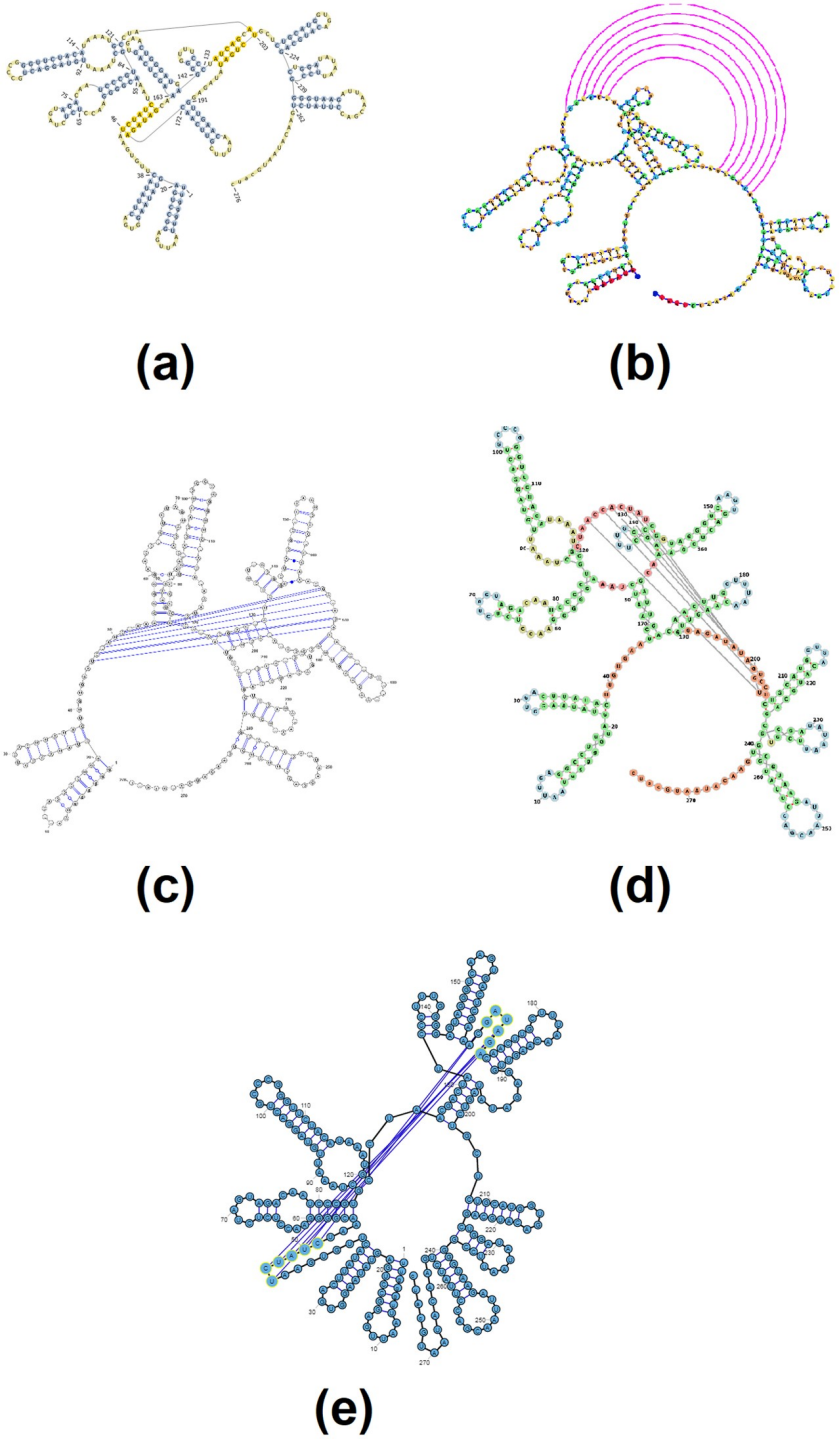
The resulting visualization for the td group I intron pseudoknot, labeled as [AB] by jViz.RNA 4.0. Structure taken from http://pseudoviewer.inha.ac.kr/, examples tab (Accession ID: LC074723.1) images produced utilizing: (a) **PseudoViewer 3** [23], (b) **jViz.RNA 4.0**, (c) **VARNA** [29], (d) **Forna** [31], and (e) **RiboSketch** [30].

**Fig 7:**
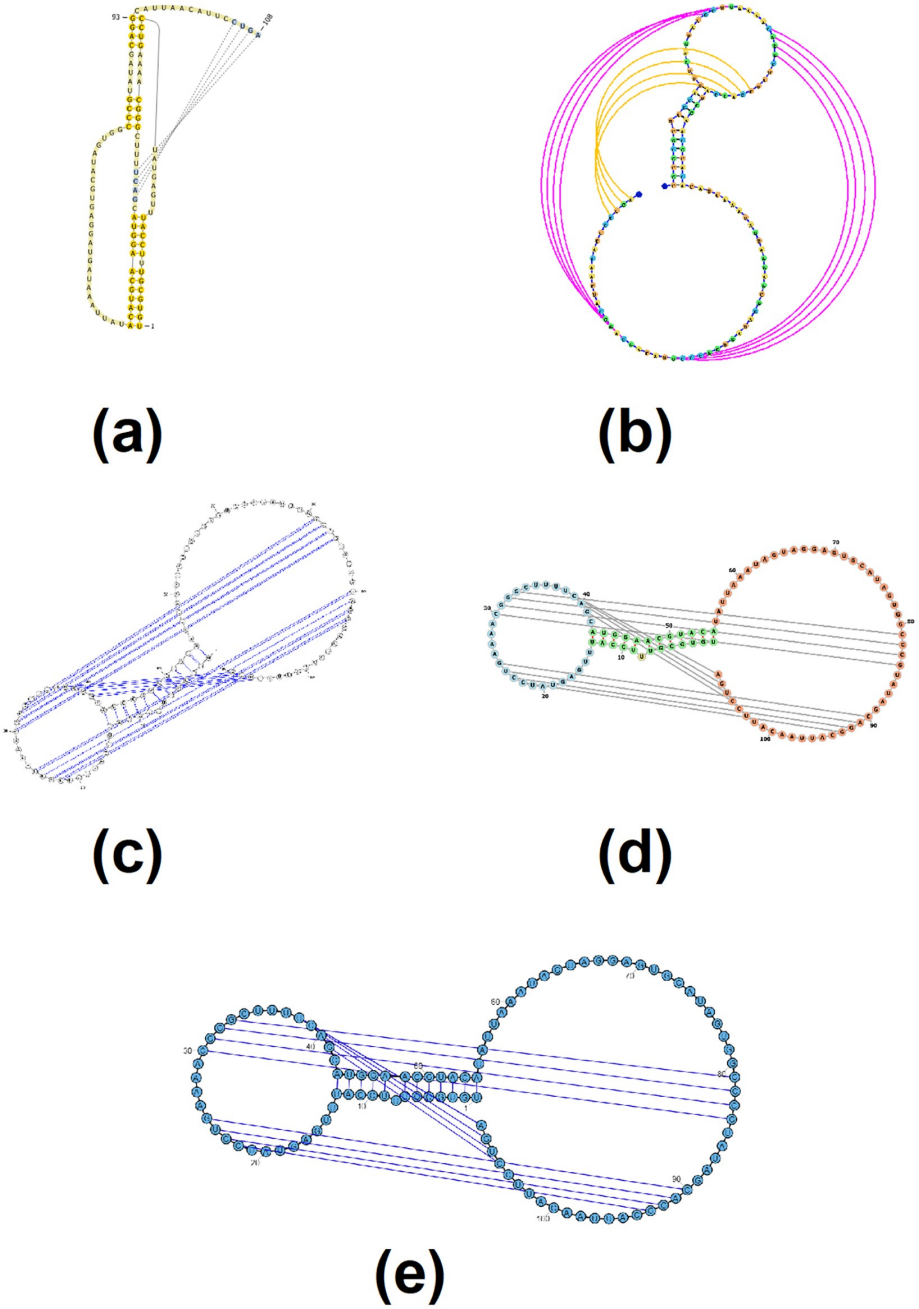
The resulting visualization of *E. coli* alpha pseudoknot, labeled [ABC] by jViz.RNA 4.0 (EMBL number X02543) [39] utilizing: (a) PseudoViewer 3 [23], (b) jViz.RNA 4.0, (c) VARNA [29], (d) Forna [31], and (e) RiboSketch [30].

Figs 3–7 demonstrate the visualization capacities which were added to jViz.RNA 4.0. Figs 4–7 compare jViz.RNA 4.0 to the four other dynamic RNA visualization software mentioned in the Introduction.

RNA visualization software can be roughly divided into three categories; The first category includes software such as VARNA [29], RiboSketch [30], and Forna [31]. These software allow for pseudoknot visualization, but make no explicit attempt to detect them or handle them. The second category includes software such as RNApdbee [18] and R-Chie [20]. These software detect pseudoknots and provide some degree of specialized pseudoknot visualization using colours. The final category of RNA visualization software includes jViz.RNA 4.0 and PseudoViewer [23]. These software offer a specialized visualization of pseudoknots, allow for user interaction with the structure layout, and take a specialized approach which entails an attempt to reduce overlap between the pseudoknot base-pairs and the non-pseudoknotted base-pairs.

The main difference between jViz.RNA 4.0 and PseudoViewer [23], however, lies in the fact that jViz.RNA 4.0 attempts to draw the pseudoknot around the main structure of the RNA, while PseudoViewer [23] draws the pseudoknotted regions first and places the structure model around them. Additionally, users can, if they need to, modify the positioning of pseudoknots by interacting with “helper nodes,” a set of dynamic bodies designed to provide interactive control over pseudoknot placement (Fig 3).

Figs 4 and 5 demonstrate the visualization results for rather simple pseudoknots. The difference between jViz.RNA 4.0 and PseudoViewer [23] is immediately evident. While PseudoViewer [23] has a predetermined method to visualize pseudoknots, and then lays out the remainder of the structure around the pseudoknotted elements, jViz.RNA 4.0 lays out the main structure and the pseudoknots around it. This mode of operation makes it easier to recognize structural elements.

Figs 6 and 7 further demonstrate the advantages jViz.RNA 4.0 offers as structures or pseudoknots become more complex. The visualization produced by PseudoViewer [23] makes it difficult to visually classify both pseudoknot type, and some of the RNA structural elements. Conversely, jViz.RNA 4.0 produces a visualization which allows for quick classification of the pseudoknot complexity level and type, as well as which structural elements it joins. The visualizations produced by VARNA [29], Forna [31], and RiboSketch [30] allow for the identification of RNA structural elements as well, however they do not induce a quick identification of the pseudoknot complexity. This is particularly visible in Fig 7.

Furthermore, Fig 7 demonstrates that as the pseudoknot complexity increases, even PseudoViewer [23] may resort to adding pseudoknots on top of its structure layout without a specialized approach for higher levels of complexity. On the other hand, jViz.RNA 4.0 maintains the same method for pseudoknot visualization, but introduces a color code which allows pseudoknots and their associated complexity to be easily spotted by users of the software, an idea similar to what is employed in RNApdbee [18] and R-chie [20].

### RNA interactive editing

**Fig 8:**
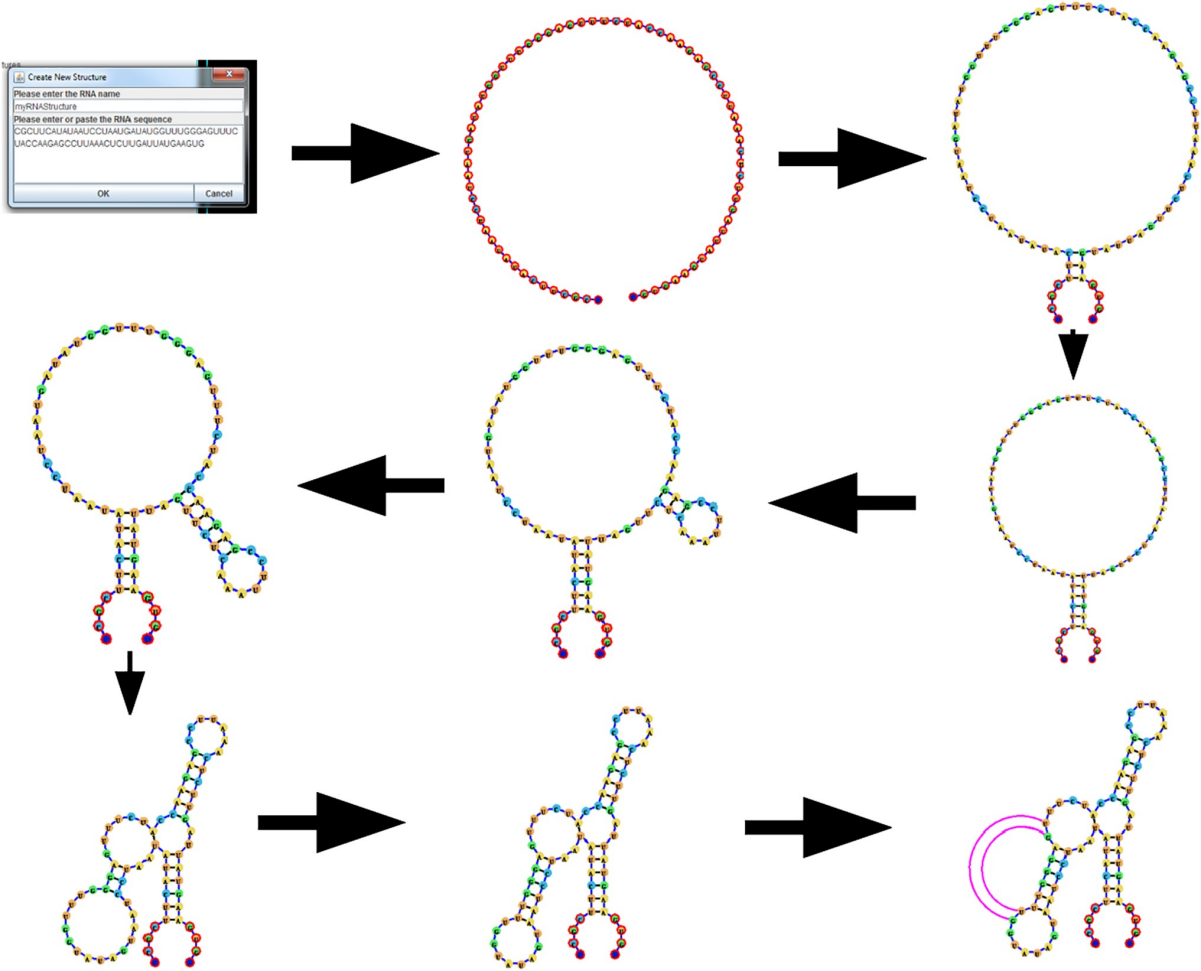
An example of the process involved in creating an RNA structure using jViz.RNA 4.0. Users can feed the sequence into the software, and introduce base-pairs between the required bases. While not shown, users can also remove base-pairs as easily as they can add them.

Fig 8 demonstrates the RNA interactive editing capabilities added to the current iteration of jViz.RNA [32]. Utilizing the RNA editing feature users can input an RNA sequence into jViz.RNA, and build a custom RNA structure of arbitrary complexity. The sequence begins as a structure with only one node. As users add or remove base-pairs, additional base-pair and loop nodes are added.

Additionally, users can introduce pseudoknot base-pairs into the structure. With the addition of every base-pair, Algorithm 1 is called on the structure. Thus, when a pseudoknot base-pair, i.e. a base-pair between two nucleotides from two different loops, is introduced, jViz.RNA 4.0 correctly labels the base-pair and creates the appropriate visualization.

As mentioned, users can further manipulate the pseudoknotted base-pairs using the helper nodes which control the pseudoknot arc size.

## Discussion

The work presented in this manuscript describes an extension of jViz.RNA 3.0 [32] by two features which place it as one of the most versatile RNA visualization software available.

First, the capability to visualize a diverse set of pseudoknots has been added. While other RNA visualization tools offer pseudoknot visualization capabilities, they allow very little, if any, user interaction with the pseudoknot base-pairs such that the visualization produced by other RNA visualization tools are immutable (i.e. they cannot be modified). On the other hand, jViz.RNA 4.0 gives users a certain degree of flexibility to decide the positioning of pseudoknotted structures within the RNA visualization canvas. In addition, the use of a force based localization method for the helper nodes ensure the majority of an aesthetic layout generation is taken care of by the software. This feature can be extremely important when producing images for publications, which will introduce space constraints on the images. With the exception of PseudoViewer [23], other tools make no effort to draw pseudoknots with an effort to reduce the overlap between pseudoknots and the remaining structure.

Several examples of the pseudoknots which can be visualized have been reviewed in this manuscript, and these examples cover the majority of biologically known pseudoknots. The limitation of this method applies to theoretical structures which may have over 26 sets of base-pairs intertwined within a segment of the RNA structure. This is due to the use of the English alphabet as the labeling method, and the fact it only contains 26 symbols. However, the biological feasibility of those structures does not seem likely, and the theoretical uses for the visualization of such structures are difficult to imagine.

In addition, jViz.RNA 4.0 now offers users the ability to edit RNA structures and change their base-pairing interactively. Rather than modify RNA text files such as FASTA or CT, users can now introduce changes to the RNA structure and view the effect of those changes on the structure as they are introduced. The addition of this feature allows users to input an RNA sequence, and edit it into an arbitrary structure as they wish. This is especially useful for people who are using jViz.RNA 4.0 for the first time, but already have RNA structures they wish to visualize saved as image files.

The non-trivial work required to modify the underlying tree graph for the RNA structure ensures that the compressed graph can be edited and modified. The fact these modifications can be done on the compressed tree graph ensures that even large RNA structures very quickly settle into a stable conformation after any number of changes, providing a much faster user interaction with the software compared to the detailed graph which was employed by former jViz.RNA versions [32], as well as Forna [31], which is the only other software that allows **interactive** editing.

## Materials and methods

### RNA pseudoknot classification and visualization

The main goal in pseudoknot visualization incorporation was the creation of a system that visualizes pseudoknots with as little overlap over the main structure as possible, and allows for some degree of user adjustment of the visualization, since different users may require a different layout of the structure.

The process of pseudoknot visualization is composed of two main stages, similarly to the process in [40] and [20]. (1) pseudoknot identification and classification, and (2) pseudoknot layout. This is done since different pseudoknots may require different visualization methods. However, in our current iteration, jViz.RNA 4.0, pseudoknot base-pairs are drawn using a very similar method for all pseudoknot types. The main distinction that is employed is between main structure base-pairs and pseudoknot base-pairs.

#### The pseudoknot classification scheme

Pseudoknot classification becomes the first step in pseudoknot visualization. There is extensive work regarding pseudoknot visualization, and a detailed review of it can be found in [9]. However, two notable methods for pseudoknot classification relevant to this article are the one described in [41] and further developed in [42], as well as the methods proposed and employed in [17].

**Fig 9:**
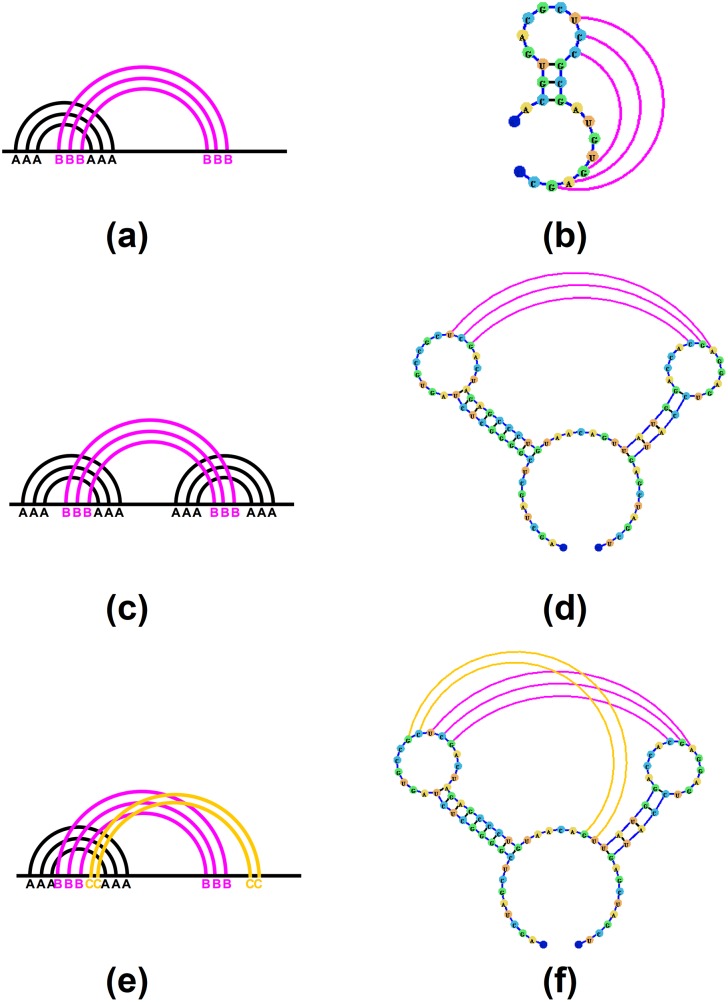
(a)-(b) A sample pseudoknotted structure which would be labeled [AB]. (c)-(d) A sample pseudoknotted structure which would be labeled [ABA]. (e)-(f) A sample pseudoknotted structure which would be labeled [ABC].

To aid in the visualization of different types (orders) of pseudoknots we propose a pseudoknot classification system. In this system pseudoknots are classified based on the number of overlapping sets of arcs each component of the RNA structure contains, similarly to the methods proposed in [40] and employed in [17, 18]. A non-pseudoknotted structure will have no overlapping arcs. While a simple H-type pseudoknot containing only one set of overlapping arcs will be labeled as an [AB] pseudoknot (Fig 9a and 9b), and a kissing hairpin pseudoknot (named so for having a set of base-pairs connecting two terminal hairpin loops) will be labeled [ABA] (Fig 9c and 9d). Each consecutive letter in the English alphabet denotes a higher level of complexity, such that a pseudoknot labeled with a label containing the letter ‘C’ will have three sets of overlapping arcs (Fig 9e and 9f).

#### RNA base-pair labeling

In the first step, the structure is considered as a graph *G* = {*V*, *E*} where each nucleotide is a vertex *v* ∈ *V* and each base-pair is an edge *e* ∈ *E*. Given this graph, the procedure for labeling base-pairs is shown in Algorithm 1.

**Algorithm 1** Base-pair Labeling

**for each** *e*_1_ ∈ *E* **do**        ⊳ edges are scanned from the 5’ to 3’ direction

 create a list *IntersectingBonds* = {}

 **if** *e*_1_ is labeled with NULL **then**

  **for each** *e*_2_ ∈ *E* **do**

   **if** (*e*_2_ and *e*_1_ intersect) AND (*e*_2_ is **not** labeled NULL) **then**

    add *e*_2_ to *IntersectingBonds*

   **end if**

  **end for**

  **if** *IntersectingBonds* is empty **then**

   label *e*_1_ as ***A***

  **else**

   ***label*** ← find the first available label in the list *IntersectingBonds*

   label *e*_1_ as ***label***

  **end if**

 **end if**

**end for**

Most of the algorithm is straightforward, but the statement “***label*** ← find the first available label in the list *IntersectingBonds*” requires further clarification. The pseudo-code for finding the first available label is shown in Algorithm 2:

**Algorithm 2** Find First Available Label

accept a list *IntersectingBonds*

create a list *BondsLabels* = {}

create an ordered list *Labels* = {′*A*′, ′*B*′, ′*C*′, ′*D*′, …, ′*Z*′}

**for each** *e*_1_ ∈ *IntersectingBonds* **do**

 **if** *e*_1_.*label* ∉ *BondsLabels* **then**

  add *e*_1_.*label* to *BondsLabels*

 **end if**

**end for**

**for each** *label* ∈ *Labels* **do**

 **if** *label* ∉ *BondsLabels* **then**

  return label      ⊳ return the first label that isn’t in *BondsLabels*,

             ⊳ even if there are higher labels in *BondsLabels*

 **end if**

**end for**

So if the set of intersecting base-pairs contains the labels {A,B,C,D}, then the first available label is ***E***. However, if the list contains the labels {A,B,D}, then the first available label is ***C*** even though a higher label, ***D***, is within the set of intersecting arcs. The highest label present within a single pseudoknot structure denotes the complexity of the pseudoknot, therefore a proper labeling of each set of arcs is essential to convey the correct complexity of the RNA structure. While the work in [40] aims to optimally define the main structure and pseudoknots in a way which minimizes the length of pseudoknotted stems, jViz.RNA 4.0 takes a simple approach which discounts optimality in favour of computational speed for the iterative process.

When all edges *e* ∈ *E* have been labeled, the structure base-pairs are divided in the following way: each base-pair (edge) with the label ***A*** is a main structure base-pair, while each base-pair with the label ***B*** or higher is a pseudoknot base-pair.

**Fig 10:**
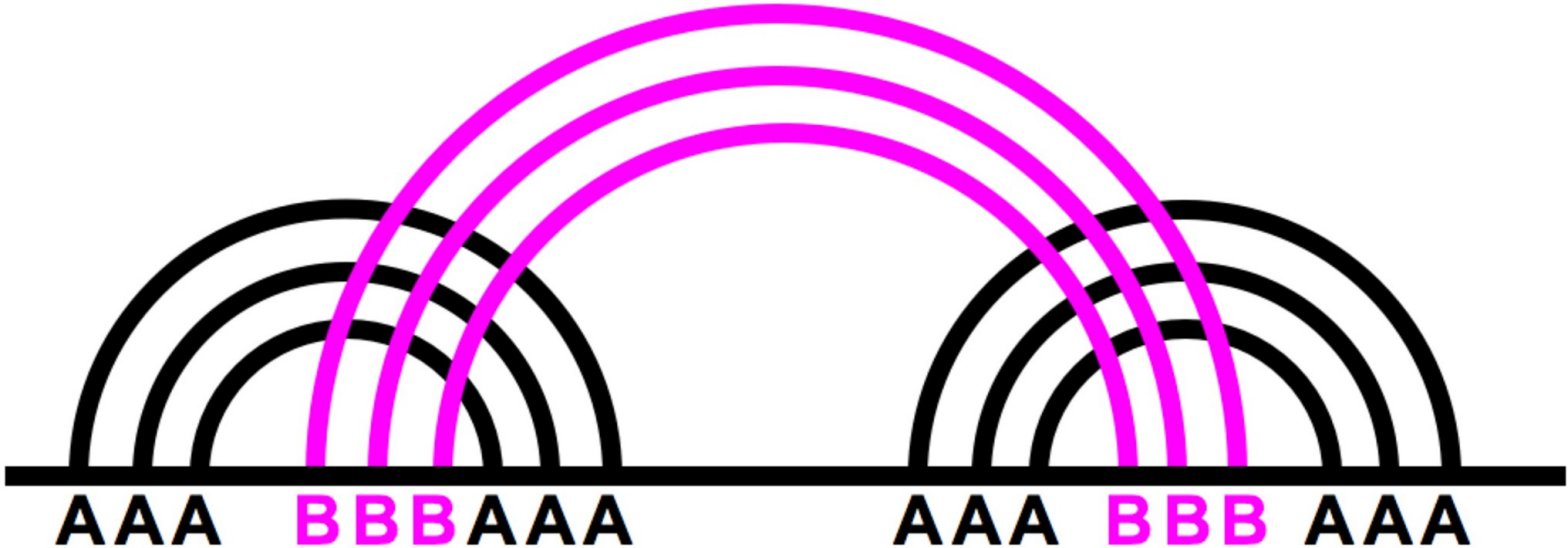
A sample pseudoknotted structure (kissing hairpin) where base-pairs have been divided into *A* and *B* base-pairs.

After all base-pairs have been labeled and divided into main structure and pseudoknotted base-pairs (e.g. Fig 10), the underlying graph model is constructed. The process for building the main structure graph is described in [32]. Therefore, for brevity purposes we will focus on the visualization of the additional pseudoknotted base-pairs.

Once the structure has had its base-pairs clearly labeled, the base-pairs can be divided into ‘main structure base-pairs’ and ‘pseudoknotted base-pairs.’ All base-pairs labeled A will be part of the main structure, while all base-pairs labeled with B or higher, will be pseudoknotted base-pairs. Additionally, all base-pairs labeled as B and higher will have a distinct color based on their letter label. This ensures a very quick visual inspection of the structure which can inform users on its complexity, and the nature of the pseudoknots it contains.

#### Pseudoknot visualization

**Fig 11:**
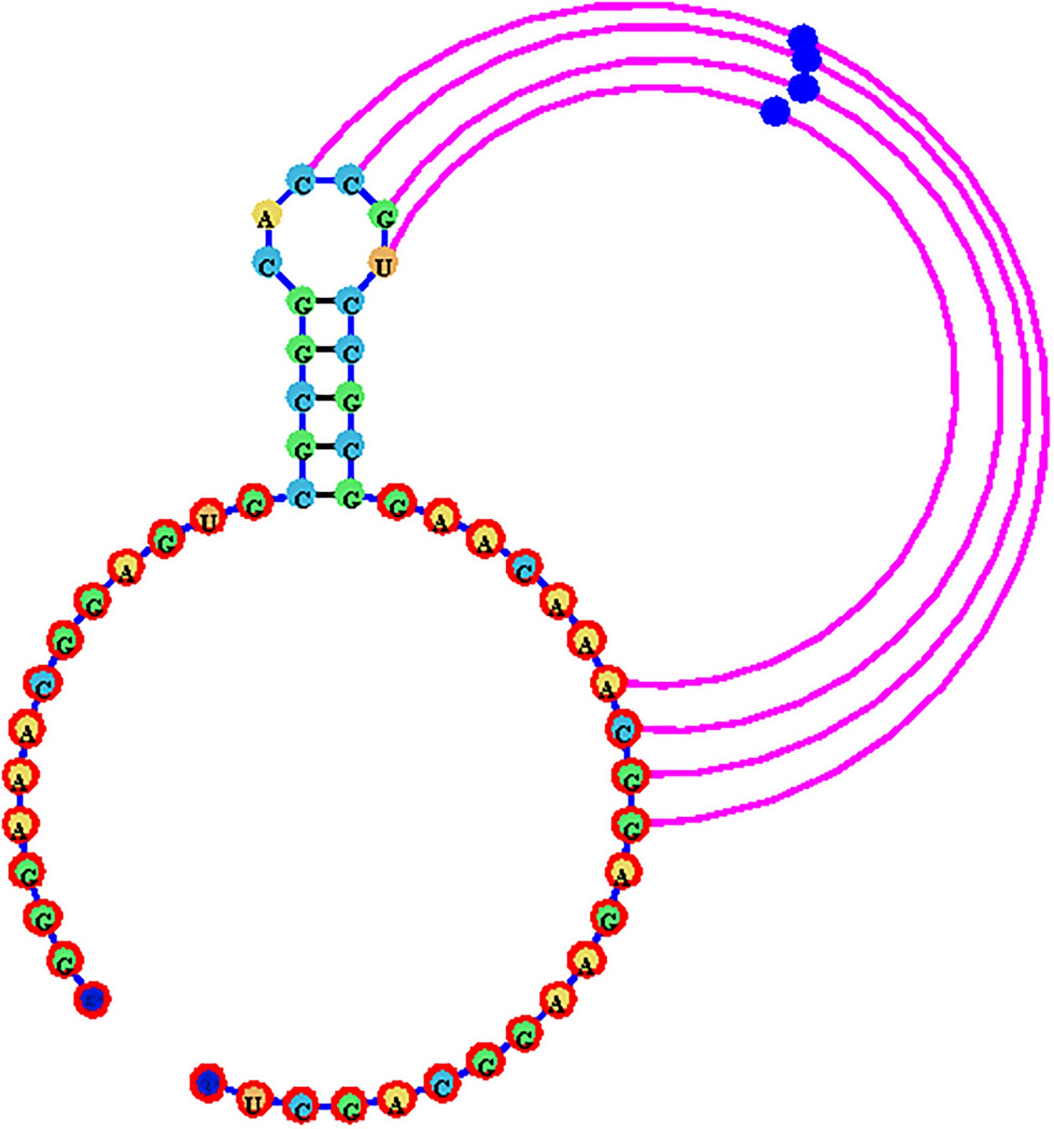
A sample visualization of the Beet Western-Yellow Virus pseudoknot (EMBL number X13063) using the novel method in jViz.RNA 4.0. Main structure base-pairs are black while pseudoknotted base-pairs are pink.

Unlike other RNA visualization software, jViz.RNA 4.0 utilizes a novel and unique pseudoknot visualization mechanism which incorporates both an automatic layout algorithm, while allowing user interaction using a distinctive control element called **helper nodes** The visualization process takes the base-pair labels into account. First, the structure is constructed as a compressed graph representing the main structure utilizing only base-pairs labeled as A. This gives an RNA model with an underlying tree graph representation. The pseudoknotted base-pairs are then added as circular arcs drawn around the structure, where each pseudoknotted base-pair is connected to two nucleotides (the nucleotides between which the base-pair occurs in the RNA structure) and its layout is controlled by a **helper node**. The helper node is a movable object which users can interact with in order to modify the outline of the pseudoknot base-pairs. This allows users to control the amount of space pseudoknotted base-pairs take up if any uses for the image require conservation of space. Fig 11 demonstrates the resulting visualization described here.

The pseudoknotted base-pairs created this way can dynamically move with the structure, or be locked into place, in a similar manner to nodes making up the main structure. This allows users to arrange each portion of the structure to their needs, without the movement of one structure element affecting another.

#### Pseudoknot base-pair visualization using helper nodes

Each pseudoknotted base-pair is a base-pair between two nucleotides *n*_*i*_ and *n*_*j*_, which each have respective positions in 2D space, ${\vec{p}}_{i}=\left[x_{i},y_{i}\right]$ and ${\vec{p}}_{j}=\left[x_{j},y_{j}\right]$, as well as positions within the structure, *i* and *j*. In order to draw a circular arc between *n*_*i*_ and *n*_*j*_, a third position ${\vec{p}}_{h}$ is computed and a helper node *n*_*h*_ is placed there. In order to compute ${\vec{p}}_{h}$, a midpoint between ${\vec{p}}_{i}$ and ${\vec{p}}_{j}$, ${\vec{p}}_{0}$, is computed, and the vector ${\vec{p}}_{r1}={\vec{p}}_{j}-{\vec{p}}_{0}$ is computed. The vector is then rotated by 90° counterclockwise around the point ${\vec{p}}_{0}$ to produce ${\vec{p}}_{r2}$, and the initial position of the helper node ${\vec{p}}_{h}$ is calculated as ${\vec{p}}_{h}={\vec{p}}_{0}+{\vec{p}}_{r2}$.

**Fig 12:**
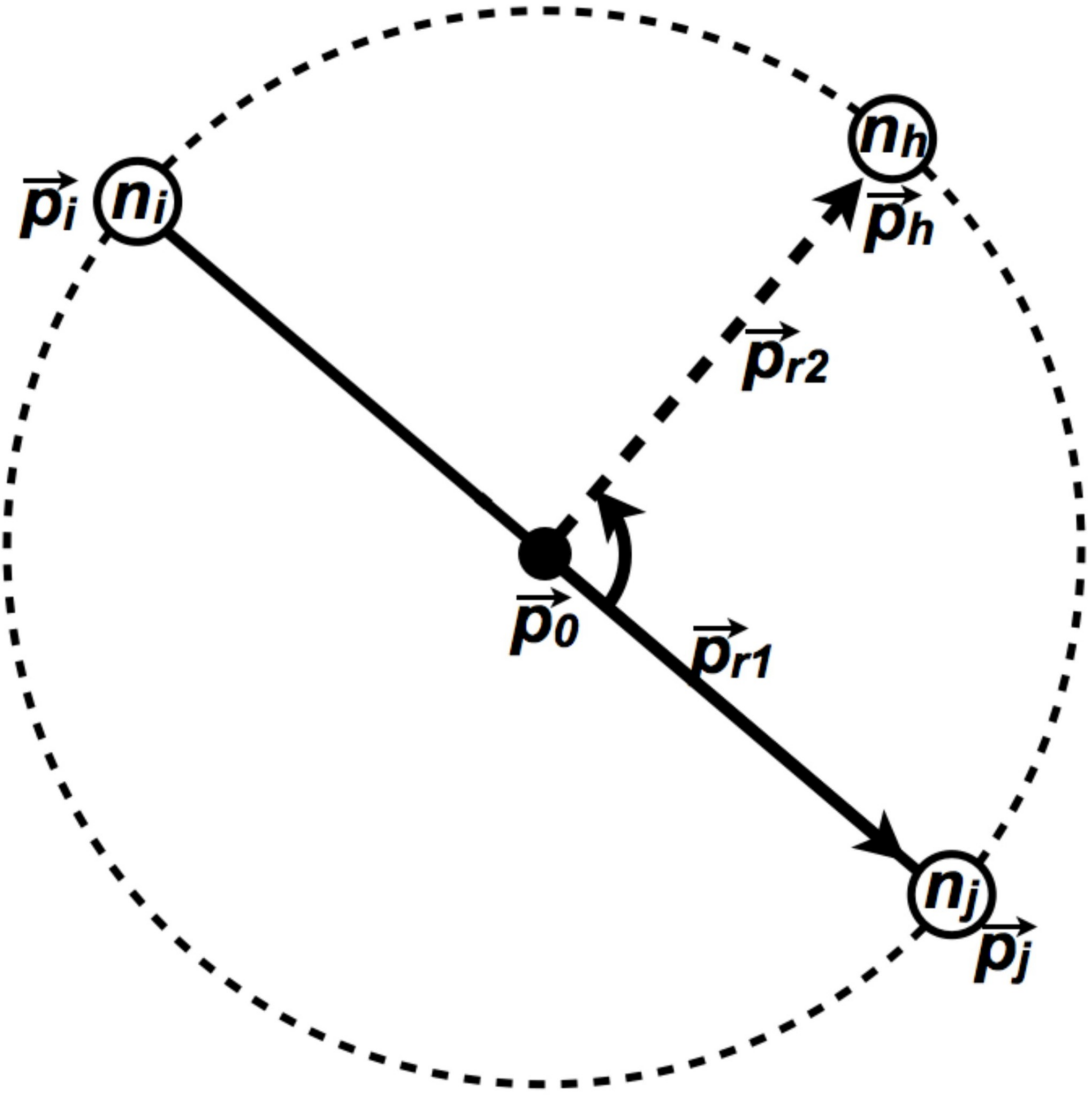
The nucleotides *n*_*i*_ and *n*_*j*_, which are joined by a pseudoknot base-pair are used to determine the position of the midpoint ${\vec{p}}_{0}$. The vector ${\vec{p}}_{r1}$ is then calculated, and rotated to produce ${\vec{p}}_{r2}$. The initial position of the helper node *n*_*h*_ is then placed at ${\vec{p}}_{h}={\vec{p}}_{0}+{\vec{p}}_{r2}$.

When the helper node position, ${\vec{p}}_{h}$ is known, the three points ${\vec{p}}_{i}$, ${\vec{p}}_{j}$, and ${\vec{p}}_{h}$ can be used to calculate a single circle through which the ciruclar arc of the pseudoknotted base-pair will be drawn (Fig 12).

However, such an initial layout of the point ${\vec{p}}_{h}$ can cause the circular arc to still occlude other structural elements, or alternatively the arc may be stretched out too far around the structure and may not serve the user’s needs, or may conflict with limitations for space to produce publication images. To that end, the helper node *n*_*h*_ is in fact a dynamic and responsive part of the structure that is capable of movement independently of the structure.

**Fig 13:**
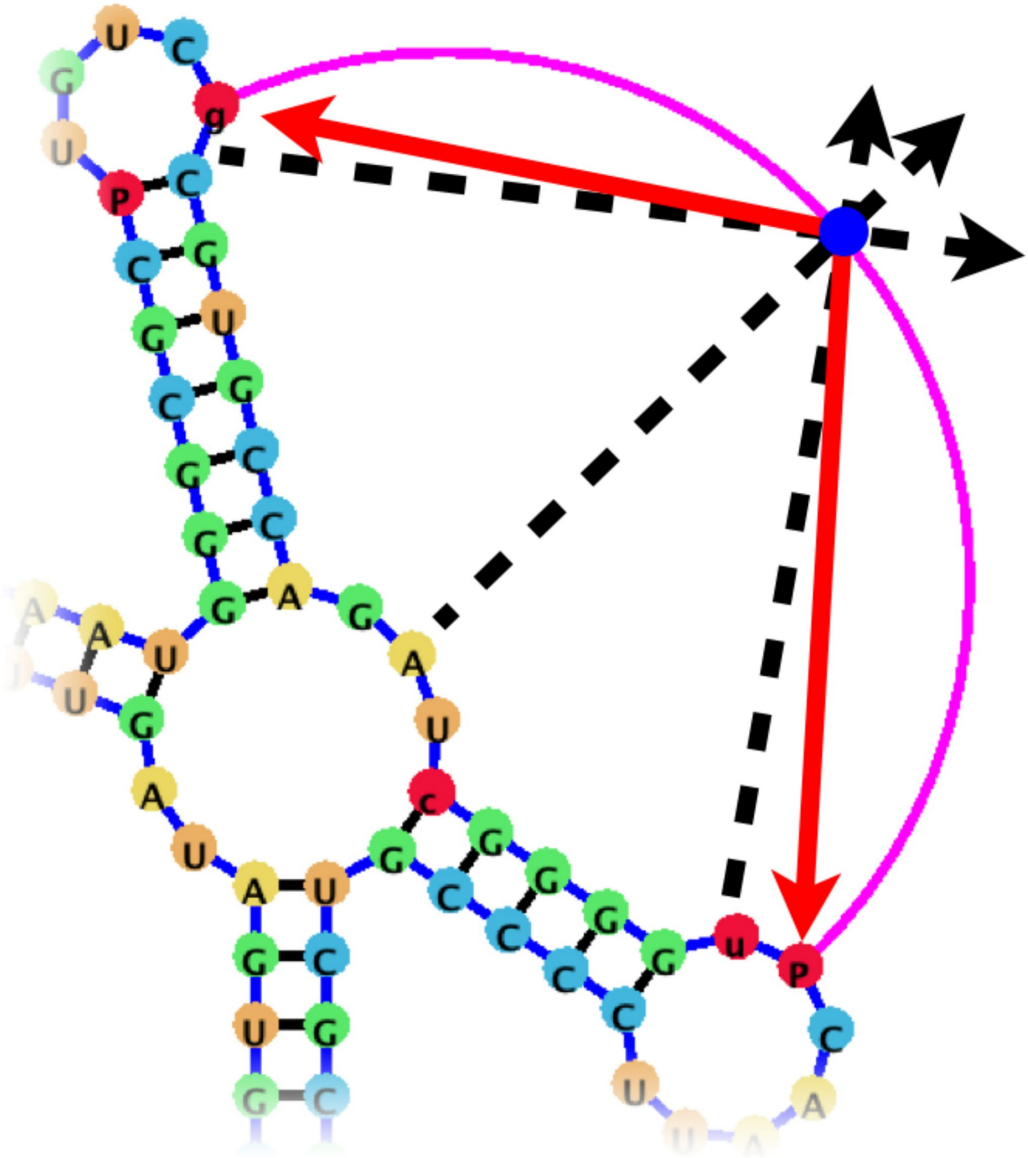
The helper node (blue sphere) is capable of independent motion since it experiences repulsion forces (black arrows) from the loop nodes in the RNA structure, and attraction forces (red arrows) from the nucleotides it creates a pseudoknot arc with.

The helper node *n*_*h*_ is designed to find an aesthetically suitable position for itself using a similar method employed for the remainder of the structure nodes [32]; Just like each node in the compressed graph of the structure is controlled by attraction and repulsion forces which allow it to find a suitable position relative to surrounding nodes, so does the helper node. It is attracted by the nucleotides which make up the pseudoknotted base-pair according to the following equation:

$$\begin{array}{c} \vec{A}=K_{h}\times \Delta \vec{d} \end{array}$$ (1)

where *K*_*h*_ is a coefficient controlling the magnitude of the force, and Δ*d* is the absolute distance between the helper node and either nucleotide (the equation is calculated for both nucleotides *i* and *j*), and repelled by the loop nodes in the structure according to the following equation:

$$\begin{array}{c} \vec{R}=\frac{G_{h}\hat{u}}{\Delta d^{2}} \end{array}$$ (2)

where *G*_*h*_ is a repulsion coefficient controlling the magnitude of the force, $\hat{u}$ is a unit vector pointing in the direction of the force, and Δ*d* is the absolute distance between the helper node and each of the loops *L* of the structure. In this manner, the helper node attempts to move away from the structure, but is drawn back by the nucleotides which make up the pseudoknot base-pair. Furthermore, the attraction coefficient *K*_*h*_, used in calculating $\vec{A}$, is itself governed by the difference in structure position between the nucleotides as follows:

$$\begin{array}{c} K_{h}=\frac{K_{h}^{\prime }}{\left|i-j\right|} \end{array}$$ (3)

where $K_{h}^{\prime }$ is the attraction coefficient all helper nodes derive their personal *K*_*h*_ from, and *i* and *j* are the positions of nucleotides *n*_*i*_ and *n*_*j*_ (between which the pseudoknot base-pair exists) within the sequence (Nucleotides can have positions from 1 to *N* where *N* is the length of the sequence). As a result, pseudoknots between nucleotides that are further apart (and thus have likely more structural components between them) are allowed to move further due to weaker attraction forces (Fig 13).

In addition to its automatic layout, the helper nodes can be controlled by the user to fit their desired visualization. The nodes can be moved by the user and locked into place like other structural components. This allows users to force the pseudoknot base-pairs to any position they may require for their purposes, as can be seen in Fig 3.

### Editing RNA base-pairs

In the context of RNA base-pair editing there are two possible actions: creating a new base pair between the unpaired nucleotides *n*_*i*_ and *n*_*j*_, or removing a base pair between the paired nucleotides *n*_*i*_ and *n*_*j*_, where *n*_*i*_ and *n*_*j*_ are paired to each other. While other software such as Forna [31] and RiboSketch [30] offer such functionality, they both utilize a detailed graph representation (Fig 2b) where nucleotides and base-pairs are mapped to vertexes and edges, respectively. Such a setup makes it easier to edit the RNA structure, but produces slower run-times for large RNA molecules. (A comparison of detailed and compressed graph run times can be found in [32]). jViz.RNA 4.0 employs a compressed graph (Fig 2c) which is stored as a tree (Fig 2d), which produces faster run times. However, introducing and removing base-pairs requires a more sophisticated manipulation of the underlying tree graph.

#### Removing an existing base-pair

**Fig 14:**
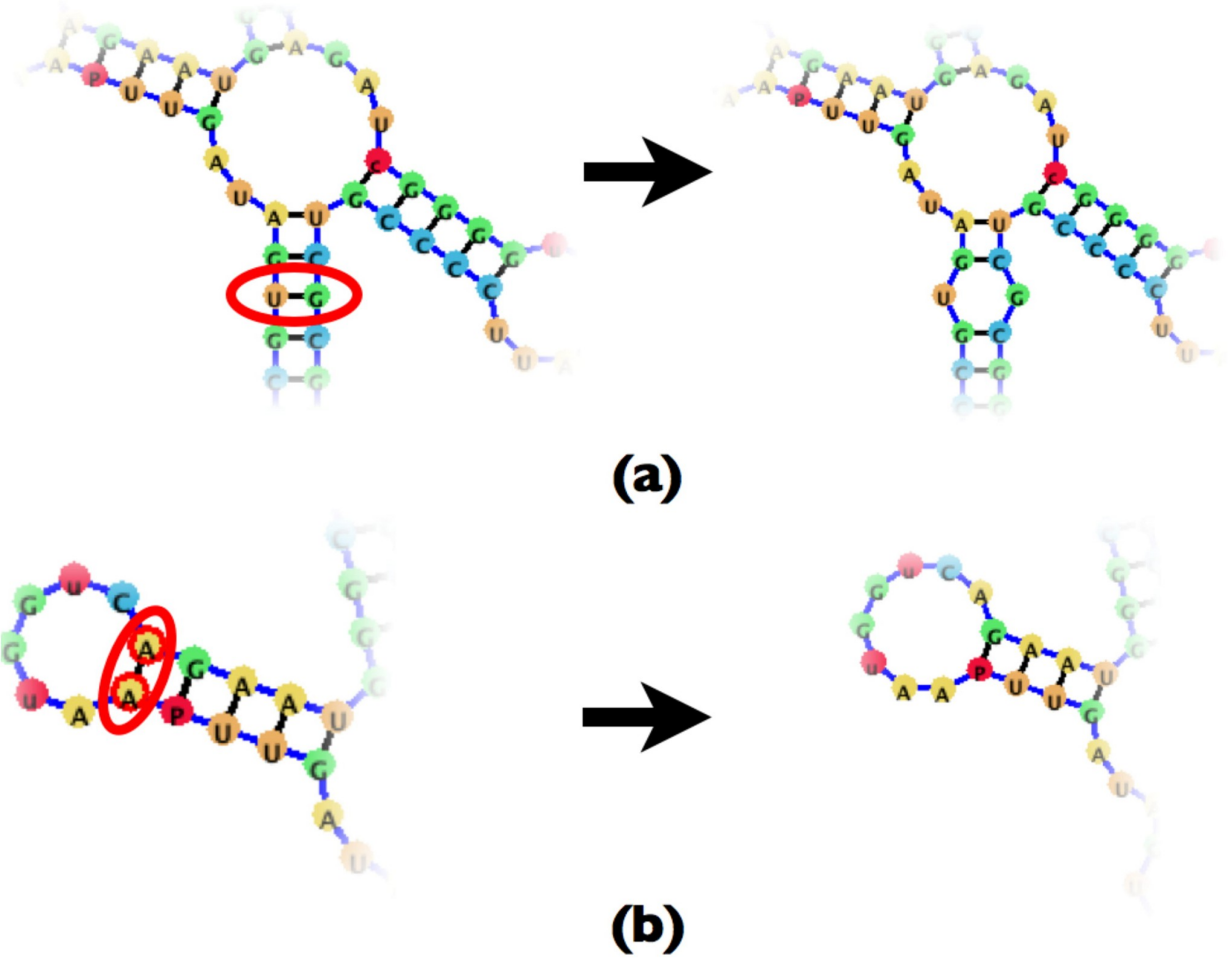
(a) An example of a base-pair deletion where both adjacent nodes are base-pairs. (b) An example of a base-pair deletion where the child node is a loop while the parent node is a base-pair.

**Fig 15:**
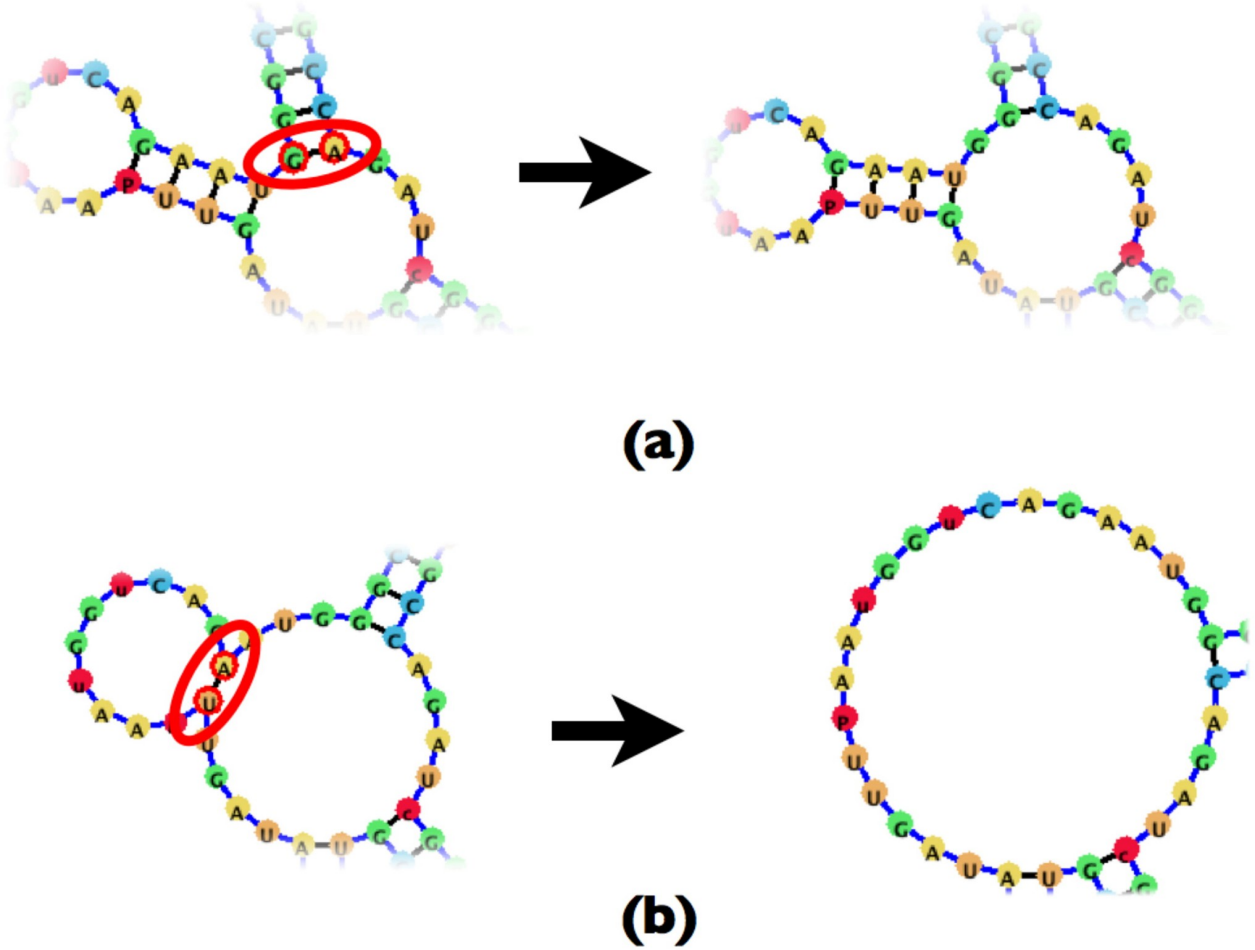
(a) An example of a base-pair deletion where the parent node is a loop node while the child node is a base-pair. (b) An example of a base-pair deletion where both adjacent nodes are loops, resulting in a combined loop.

Removing an existing base-pair requires first removing the base-pair node *B*, and replacing it with a loop node *L*. However, depending on the child (*C*) and parent (*P*) node of the base-pair node further nodes may need to be removed. Since both the parent and child nodes can be both base-pair nodes or loop nodes, there are four possible scenarios to consider, and those can be seen in Figs 14 and 15.

In essence, removing a base-pair may create a new loop, or fuse existing loops together. The difficulty which the compressed graph representation introduces is the need to account for the connectivity of the loops created by the removal of base-pairs.

#### Adding a new base-pair

**Fig 16:**
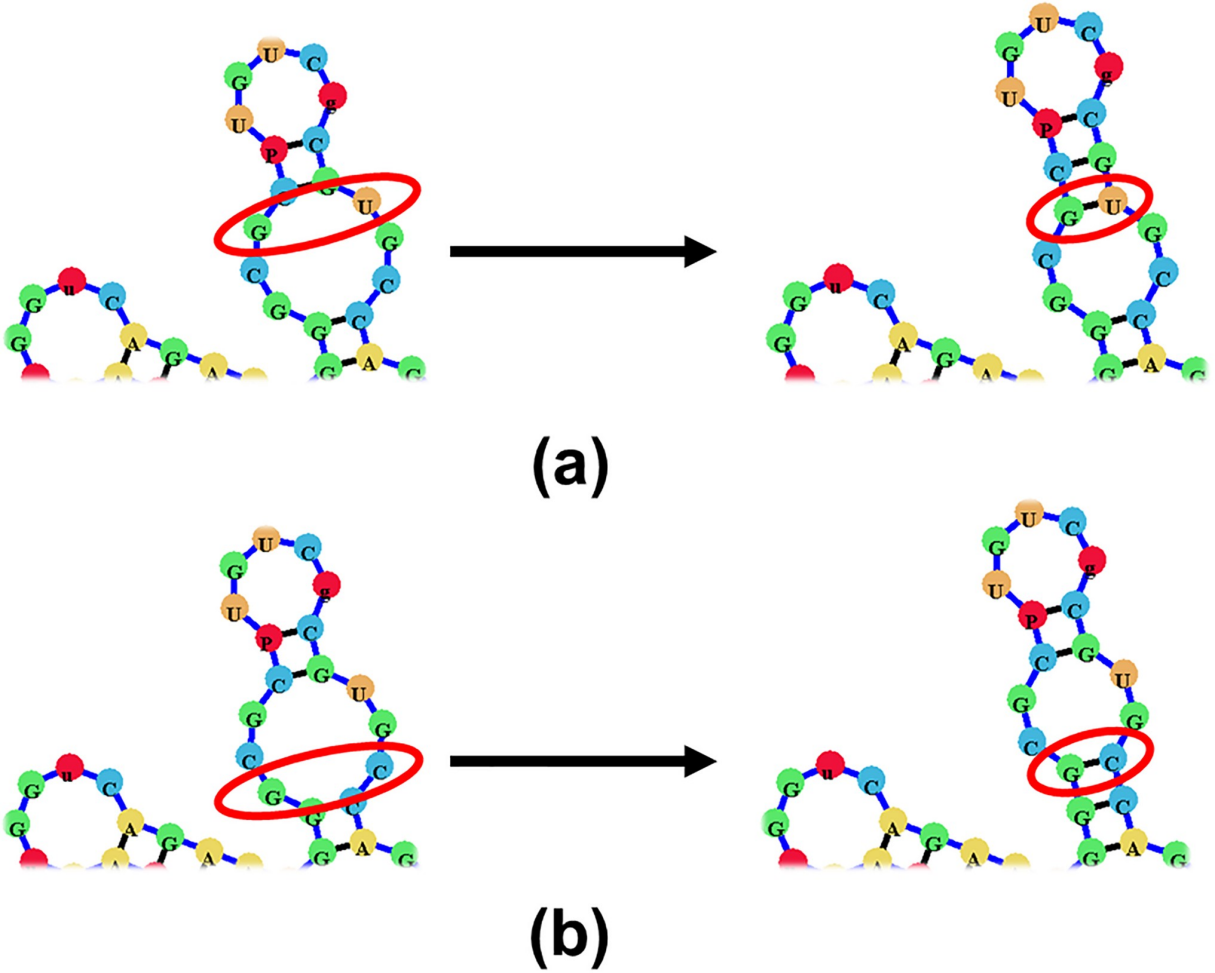
(a) An example of a base-pair addition where only the parent of the resulting base-pair is a loop node. (b) An example of a base-pair addition where only the child node is a loop while the parent node is a base-pair.

**Fig 17:**
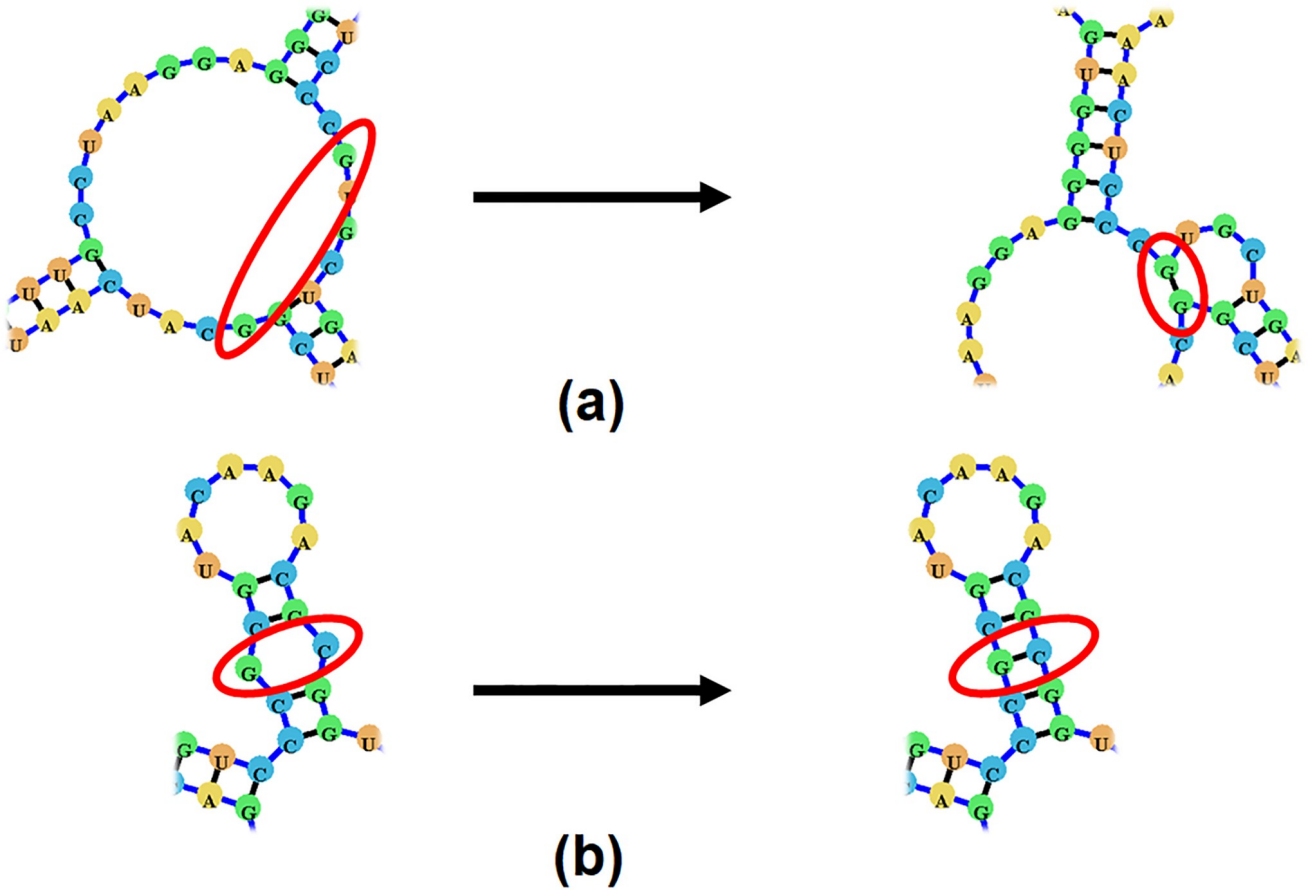
(a) An example of a base-pair addition which splits the loop node into two smaller loop nodes. (b) An example of a base-pair addition where both parent and child nodes are also base-pairs.

Adding a base-pair will always happen within the context of a loop *L*. The base-pair *B* will split the loop into two nodes and in a similar manner to removing a base-pair, adding a new base-pair to an existing structure can have four possible outcomes which are shown in Figs 16 and 17.

While removing a base-pair may fuse loops together, Fig 17a demonstrates the potential for splitting loops by the addition of base-pairs. This requires any base-pair addition, like deletion, to take into account the connectivity of the affected loop and restructure the underlying tree accordingly.

## Conclusion

jViz.RNA has been extended to accommodate visualization of the great majority of biologically classified RNA pseudoknots. Care was taken to reduce, and in many cases eliminate, the overlap between pseudoknots and the remaining RNA structure. Our novel method utilizing circular arcs and helper nodes for pseudoknot visualization leads to a very intuitive and aesthetically pleasing structure layout. In addition, the secondary structure can now be edited interactively with the underlying tree graph responding very quickly to the changes in configuration. The novelty imbued into jViz.RNA 4.0 with the implementation of these features sets it as one of the most versatile RNA visualization software available. jViz.RNA 4.0 now offers visualization capabilities for a wide array for both pseudoknotted and non-pseudoknotted RNA molecules. Furthermore, the introduction of interactive editing features allow users to modify the main RNA structure configuration, as well as introduce or remove pseudoknots to explore alternative structures, and even create an entirely novel structure starting only with the RNA sequence. Similar to previous versions, jViz.RNA 4.0 remains platform independent.

In terms of significance, jViz.RNA 4.0 now allows users to entirely customize the layout of any RNA molecule, both its structural elements and its pseudoknotted base-pairs. The work presented in this manuscript revolved around extending the usability of jViz.RNA 4.0 while maintaining a dynamic and responsive layout that can be manipulated by the user, and preserve the fast user responsiveness introduced by the compressed graph in [32]. This degree of flexibility, responsiveness, and ease of modification can allow users who come from mostly natural sciences background to begin working with jViz.RNA 4.0 without a steep learning curve.

## Supporting information

S1 Fig A striking image to be used for online publications. (TIF)

S1 File A package containing the jViz.RNa 4.0 executable, several RNA test files, and a quickstart user manual. (ZIP)
