# Supplementary figures and images for: jViz.RNA 4.0—Visualizing pseudoknots and RNA editing employing compressed tree graphs
Source: PLoS One. 2019 May 6;14(5):e0210281. doi: 10.1371/journal.pone.0210281 (PMC6502502; doi:10.1371/journal.pone.0210281)

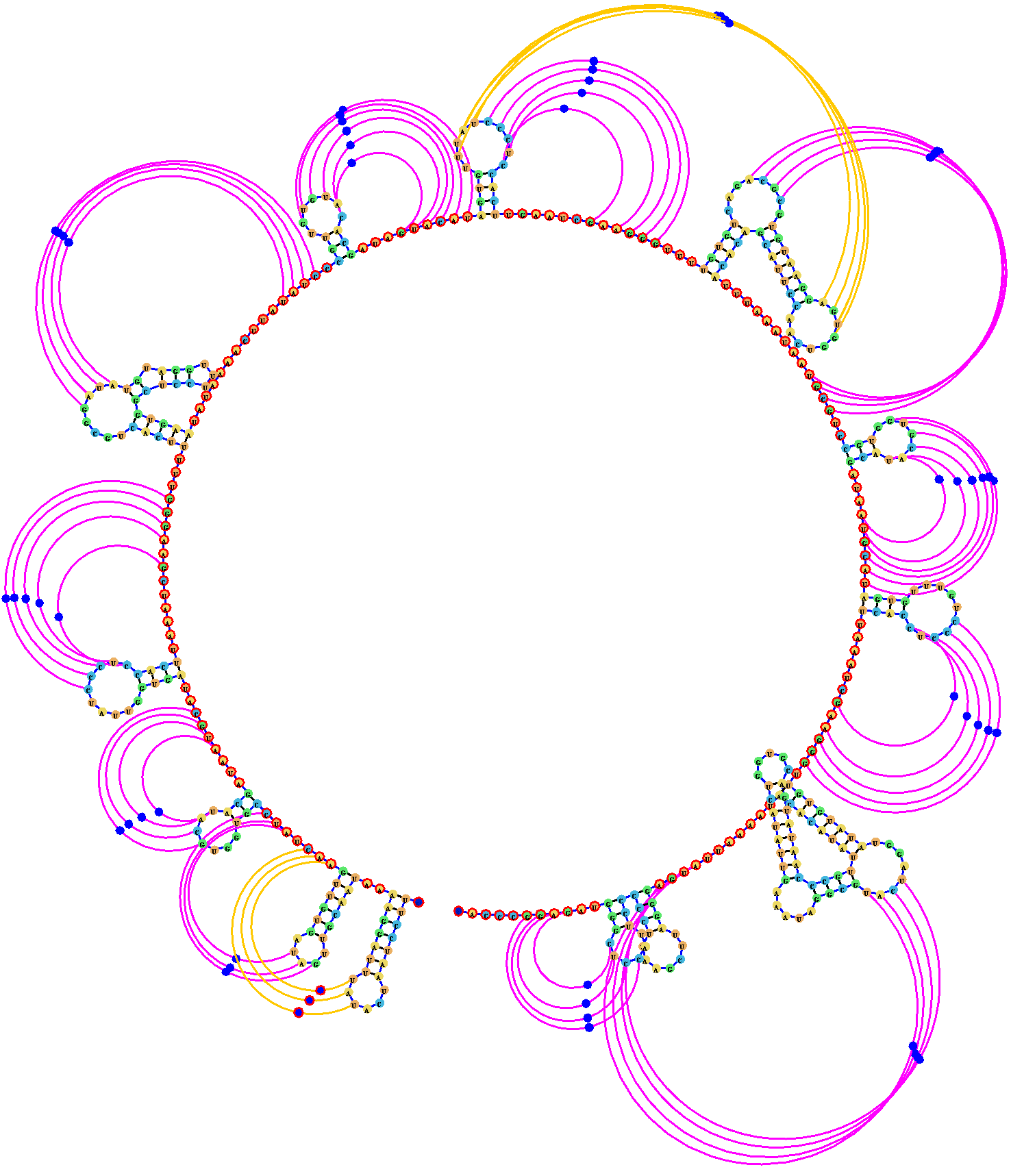

Supplement: S1 Fig — (TIF) [file pone.0210281.s001.tif]

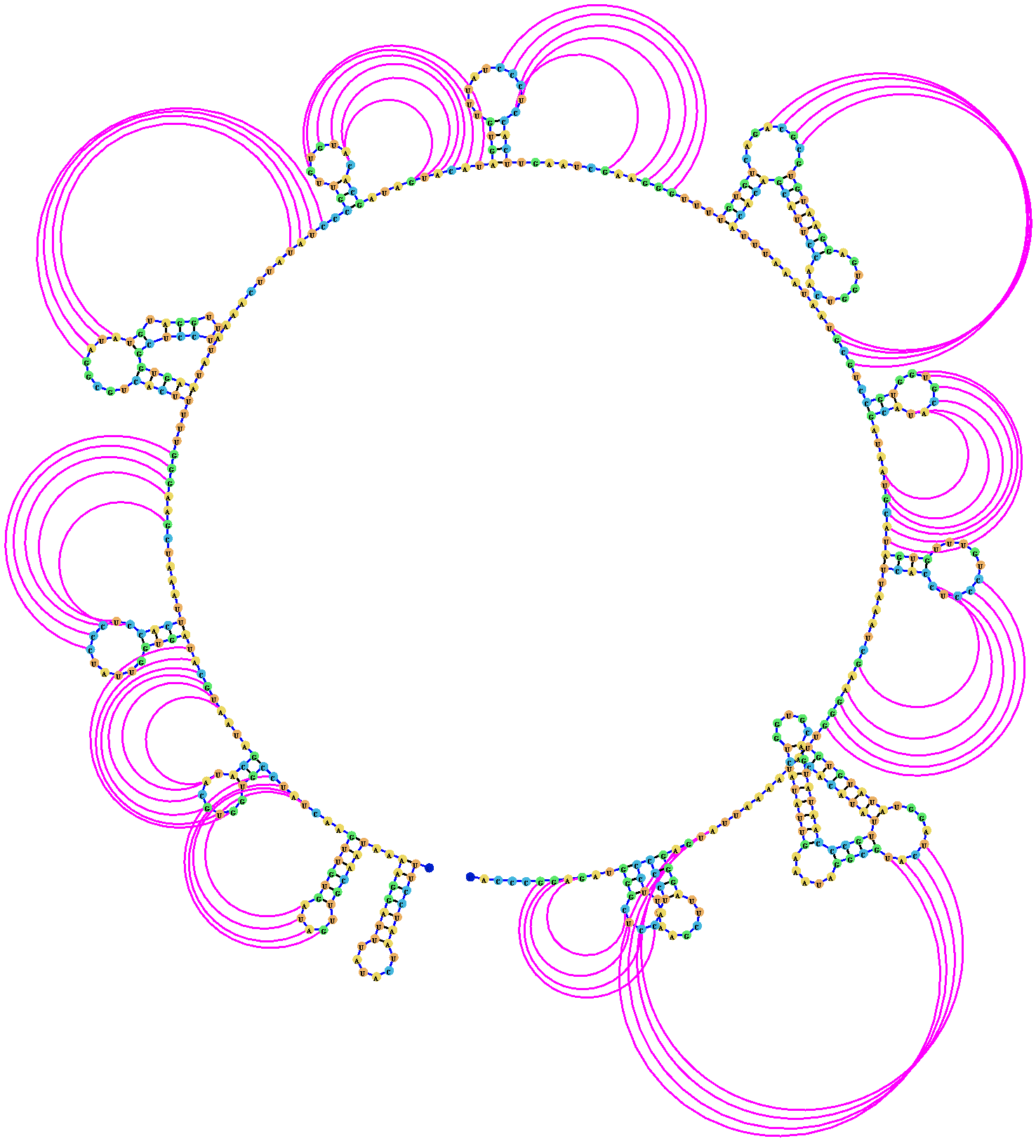

Supplement: S1 File — (ZIP) [file pone.0210281.s002.zip › jViz 4.0 Complete/Sample Images/0419_Odontoglossum_ringspot_virus_pseudoknot.png]

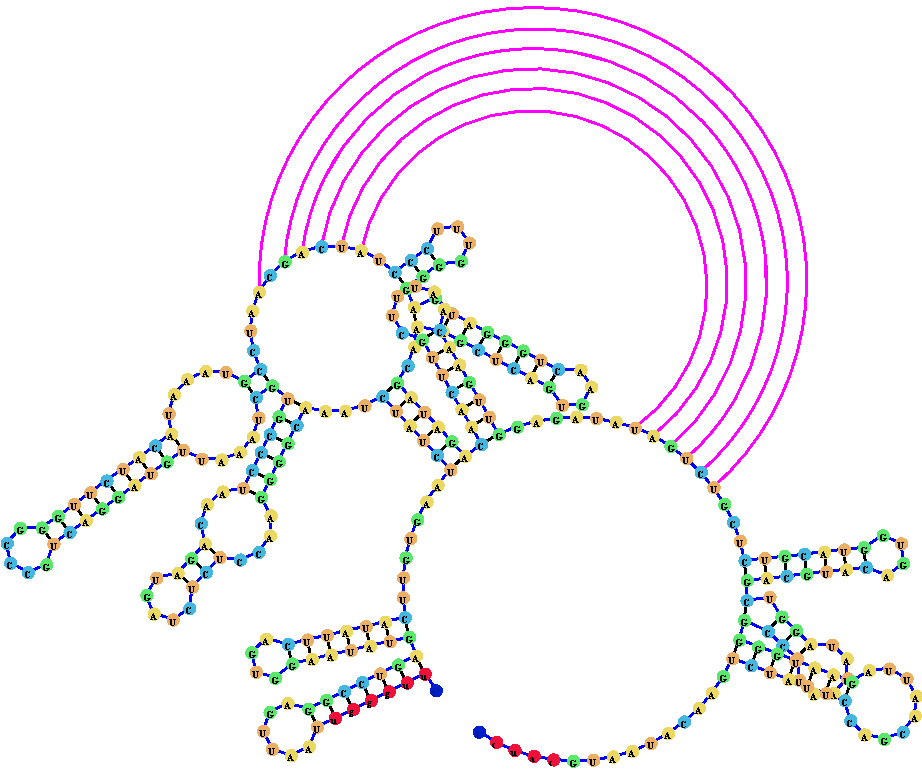

Supplement: S1 File — (ZIP) [file pone.0210281.s002.zip › jViz 4.0 Complete/Sample Images/0277_td_group_I_intron_pseudoknot.png]

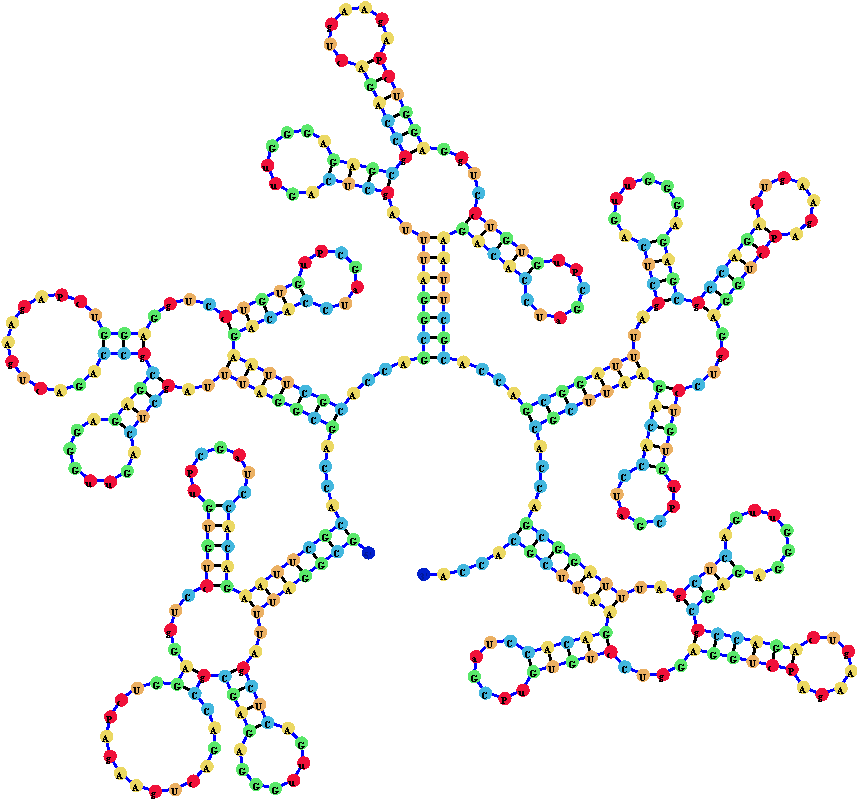

Supplement: S1 File — (ZIP) [file pone.0210281.s002.zip › jViz 4.0 Complete/Sample Images/0380_Saccharomyces_cervisiae_non-pseudoknotted.png]
