# Supplementary material for: jViz.RNA 4.0—Visualizing pseudoknots and RNA editing employing compressed tree graphs
Source: PLoS One. 2019 May 6;14(5):e0210281. doi: 10.1371/journal.pone.0210281 (PMC6502502; doi:10.1371/journal.pone.0210281)
Supplement: S1 File — (ZIP) [file pone.0210281.s002.zip › jViz 4.0 Complete/jViz 4.0 User Manual.pdf]

Simon Fraser University
School of Computing Science

jViz.RNA 4.0 Quick Start Manual

BORIS SHABASH

KAY C. WIESE

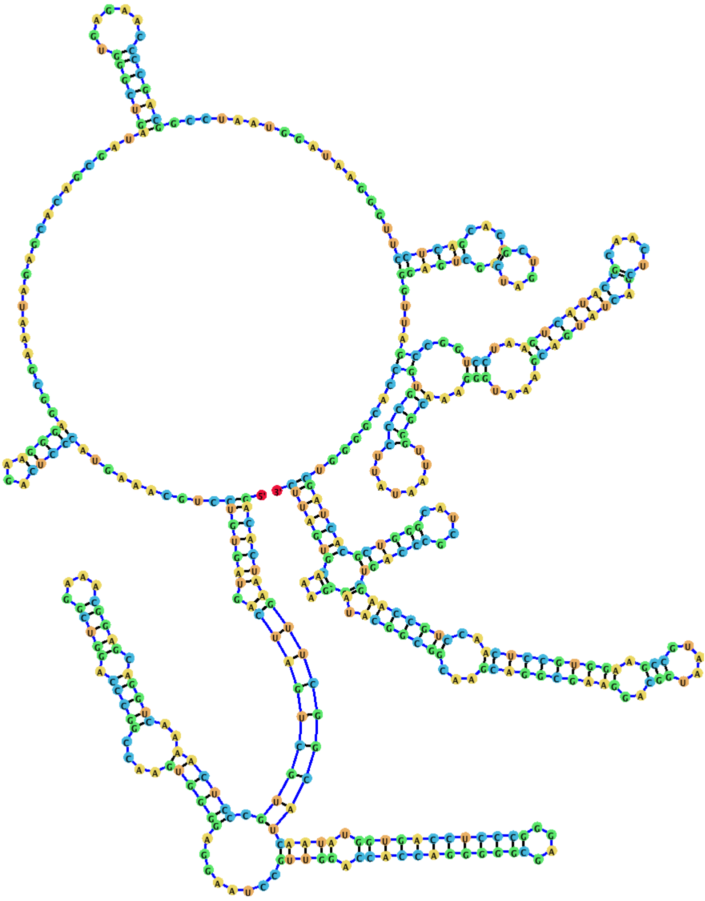

Loading RNA Secondary Structures

Before Getting Started

jViz.RNA 4.0 requires the Java environment to run. While most systems are shipped with the Java environment pre-installed, please make sure you have it by visiting https://www.java.com/en/download/help/version_manual.xml and download the latest version of Java if required (https://java.com/en/download/).

Running the software

The jViz.RNA complete package can be downloaded from https://jviz.cs.sfu.ca/download/download.html. jViz.RNA requires no installation since it is a Java `.jar` executable, only the Java Runtime environment (see above). In order to run the software, simply unzip/unpack the contents of the folder `jViz.RNA 4.0 Complete`. The folder should contain the `jViz.jar` file, and a folder of RNA secondary structures `RNA Structures`.

Running under Mac OS X and Windows:

Double-clicking the `.jar` executable launches jViz.RNA 4.0 using the default `Java` environment

Running under Ubuntu:

In order to run jViz.RNA 4.0 under Ubuntu, simply right-click on the `.jar` executable and select `Open With -> Java Runtime Environment`

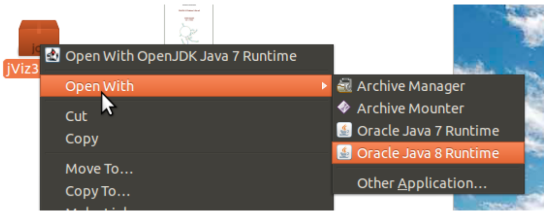

Running from the command terminal:

In Windows, Mac OS X, and Ubuntu, jViz.RNA 4.0 can be launched by navigating to the folder containing the `.jar` file, and calling the command: `java -jar jViz4.0.jar`

Visualizing the RNA structures

Double-clicking the `jViz.jar` file should launch the program. To load an RNA structure simply go to `Load File -> Load File For Structure Visualization` and select the file from the `RNA Structures` folder. The jViz.RNA software can accept .ct and .dbn/dbf files.

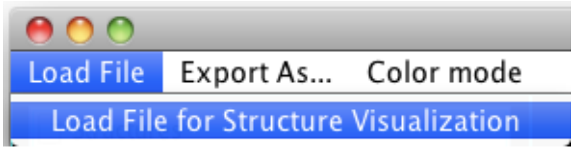

Testing the Software

We have tested the software on a wide variety of systems to confirm its stability and functionality. The tests included running jViz.RNA 4.0 on Windows 7 and 10, Mac OS X 10.6.8 and 10.11.6, and Ubuntu version 14.04.5. The software was tested under different Java versions entailing 1.7.0_77-b03, 1.7.0_80-b15, 1.8.0_91-b14, 1.8.0_102-b14 and 1.8.0_-112-b15. In total, 22 machines were tested with no faults detected. On one machine, however, we did notice an unusual behaviour where the Load file dialogue window **needed to be resized** before it renders its contents. We subsequently tested jViz.RNA 4.0 on a machine that is identical in hardware, operating system, and Java Version to the machine that produced the unusual behavior and were unable to reproduce the error. We believe that it is a machine related issue. Given our test protocol above we are very confident in the stability of jViz.RNA 4.0. However, in the unlikely event that the Load File window does not render as expected, **simply resizing it promotes proper rendering.**

Manipulating the RNA Secondary Structure

Automatic Layout

jViz.RNA uses a force based system to calculate an automatic layout for the RNA molecule. The automatic layout structure is very quick, but can still take several seconds for very large structures.

Manual Layout

Moving RNA Loops and Base-pairs

The loops and base-pairs can be manipulated by selecting them and dragging them to their desired positions. When the mouse is over a structural element, a red dashed line will indicate which element is about to be selected.

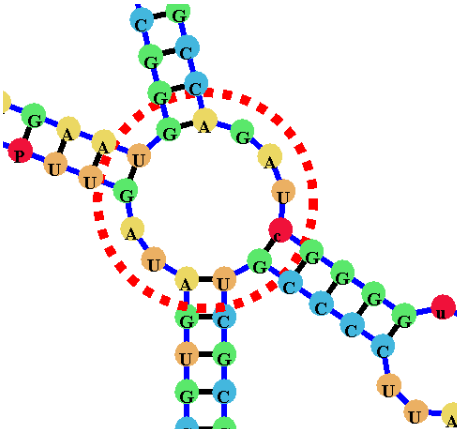

(a) *The mouse pointer is over the central node, indicating it will be selected if the user clicks it.*

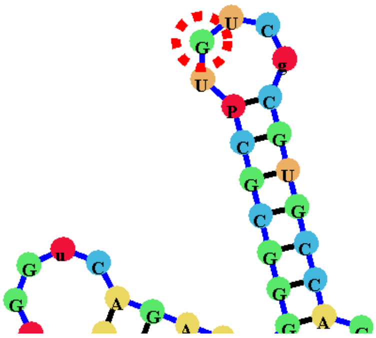

(b) *Similarly, if the mouse pointer is over a nucleotide, the nucleotide has a dashed line around it.*

Once a base-pair or loop has been selected (by single-clicking) and/or moved (by dragging), it is "locked" into its position and will not move (locked loops and base-pairs have a red hue around them). **Double-clicking** the base-pair or loop will **unlock** it, and return it to the position determined by the automatic layout algorithm. **Single-clicking** the loop basepair while it is unlocked locks it again.

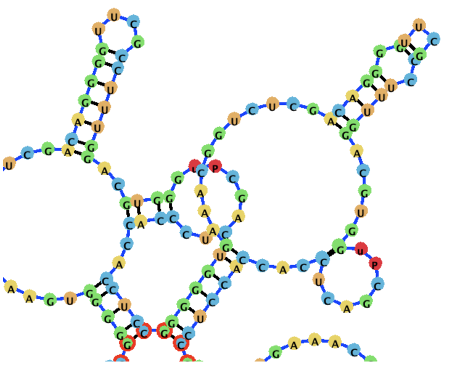

(a) *Two nodes are intersecting in the final RNA layout. They need to be separated by the user*

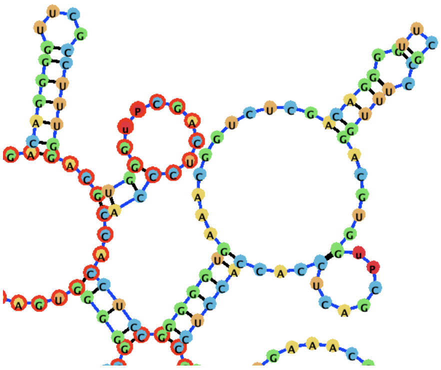

(b) *The nodes have been moved and "locked" into place (as evident by the red hue around them)*

To move an entire stem, simply move the first base-pair of the stem.

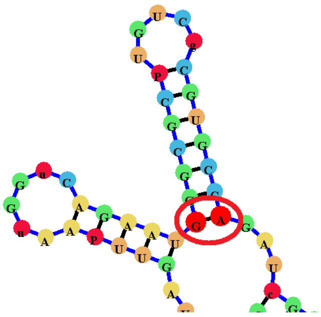

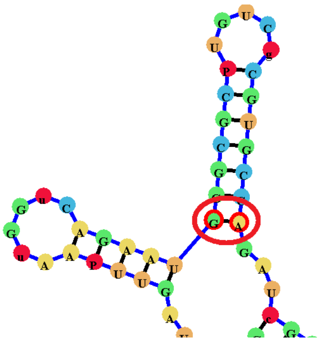

Modifying Pseudoknot Bonds

jViz.RNA 4.0 seeks to place pseudoknotted bonds such that they present minimal overlap with the main structure. However, users can manipulate the layout of pseudoknotted bonds in the same way that loops and base pairs can be manipulated. Each pseudoknotted bond has a ”helper node” associated with it, they are visible as blue spheres going through the pseudoknot bond.

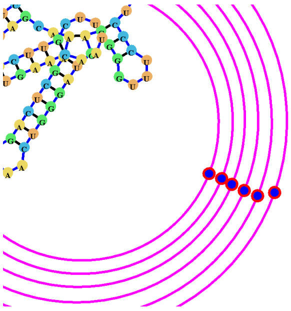

Pseudoknots can be modified by dragging the helper nodes, as can be seen in the following figure. Once selected or dragged, like loops and base-pairs, the nodes are “locked” into place (evident by a red halo around them). **They can still be moved by the user, but will not move if the structure is moved**. Double-clicking them “unlocks” them and allows them to move freely again.

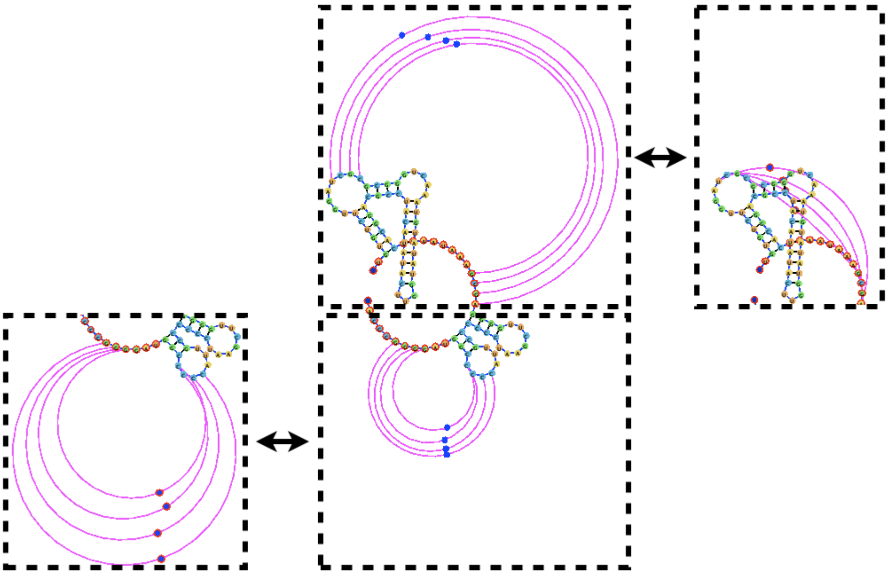

The pseudoknotted bonds can be manipulated by dragging their corresponding helper nodes.

Exporting the RNA Secondary Structure

Exporting as an image

Currently, jViz.RNA 4.0 allows for `PNG` and `EPS` file export. To export your structure as a `PNG` file, go to `Export as... -> Export as Image File -> Export as PNG Image`, or `Export as... -> Export as Image File -> Export as EPS Image`, and select a name as well as a save destination.

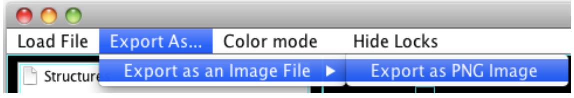

Exporting as a structure

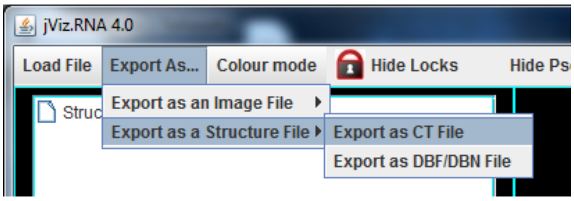

Since jViz.RNA 4.0 allows for structure editing, you can save the changes you make by exporting the structure as a `.dbn` (Dot-bracket notation) or `.ct` (ConnecT) file. To do so, go to `Export as... -> Export as a Structure File` and select the file of your choice, then select a name as well as a save destination.

Removing the locked red hue

When preparing images of the RNA molecules, it can be inconvenient for the image to contain the red hue which denotes "locked" parts of the molecule. To remove the red hue, click on `Hide Locks` at the right-most side of the top menu. To bring the red hue back, click on `Show Locks` at the same place. **Hiding the red hue DOES NOT remove the locks, it simply hides the lock indicator. To "unlock" the loops or base-pairs, simply double click them.**

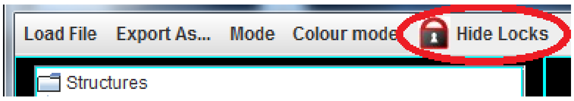

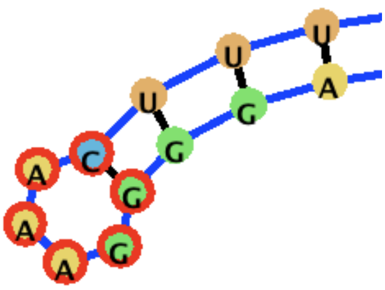

(a) *The loop has been locked into place by the user, and has a red hue to indicate it is locked*

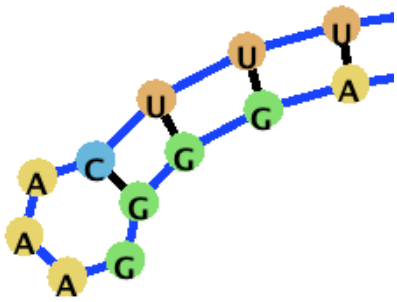

(b) *The loop is still locked. However, the red hue has been removed for visualization purposes.*

Removing Pseudoknot Helper Nodes

Similarly, the helper nodes can be removed for image export purposes by selecting the "Hide Pseudoknot Helper Node" button.

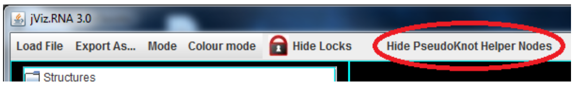

The resulting effect renders only the pseudoknot bonds, while hiding the helper nodes (However, if the nodes are locked, the pseudoknot remains locked into place and will not move with the rest of the structure).

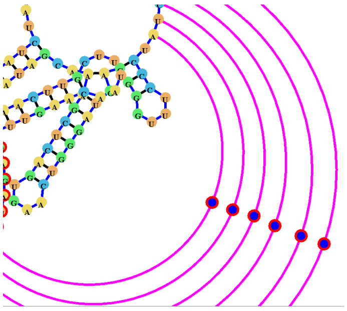

(a) *The helper nodes have been locked into place by the user, and have a red hue to indicate the lock.*

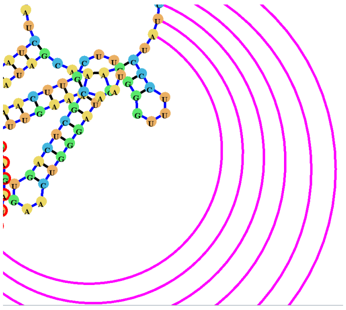

(b) *The pseudoknots are still locked. However, the helper nodes have been removed for visualization purposes.*

Editing RNA Connectivity

jViz.RNA 4.0 offers the users the unique ability to edit RNA connectivity "online," that is, using the software instead of editing the input files.

In order to remove a hydrogen bond, please select the base-pair or helper node, in the case of a pseudoknotted bond. The "Break Hydrogen Bond" button on the bottom left

will become available. Clicking this button (**or pressing 'B' on the keyboard when the button is active**) breaks the selected hydrogen bond.

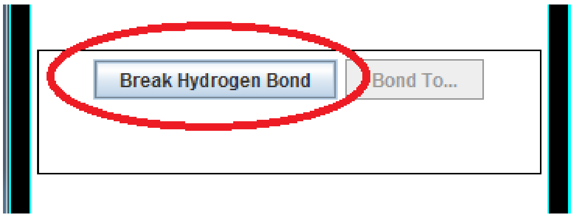

Once the hydrogen bond has been broken, the structure will adjust itself to account for the change.

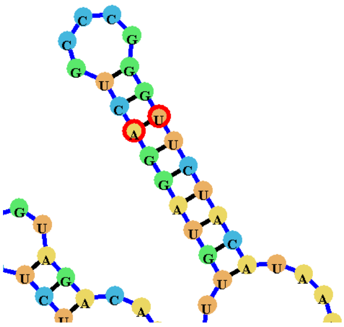

(a) *Hydrogen bond selected*

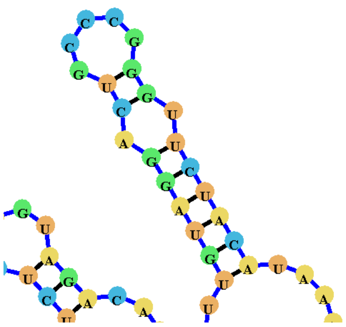

(b) *Hydrogen bond broken*

Alternatively, in order to connect two unpaired nucleotides into a base-pair, select one of the desired nucleotides. The "Bond To..." button will become available (**and can be activated by pressing 'B' on the keyboard when the button is active**) and will ask you to click the desired bond partner.

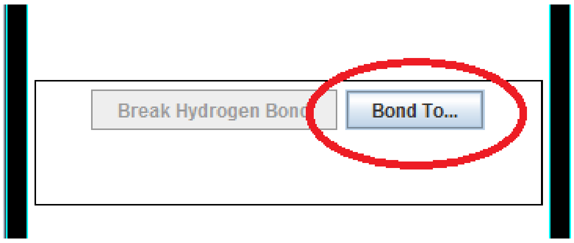

Once the desired bond partner has been selected, a new base-pair will be added to the structure. There is no limitation on which nucleotide can be bonded (so in theory, you could bond two Adenine nucleotides together). Once the new base-pair is added, the conformation of the structure could change to account for the new connectivity.

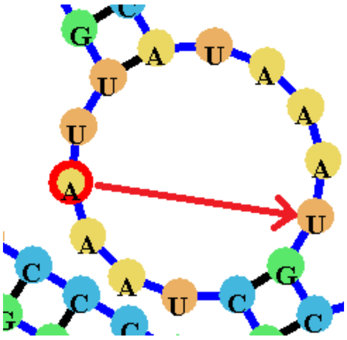

An Adenine is bonded to a Uracil, which will split the loop containing the nucleotides into two loops

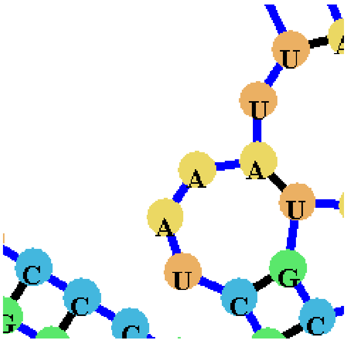

The resulting base-pair creates two new loops, causing the conformation of the structure in that area to change.

Creating a structure "from scratch"

jViz.RNA 4.0 gives you the ability to create a structure of your choice. For this you only need the RNA sequence. To do so, got to `Load File -> Make Structure Manually`. A new dialog will pop up and you will be requested to name the structure and type/paste the sequence. After the new structure has been created, you can use the structure

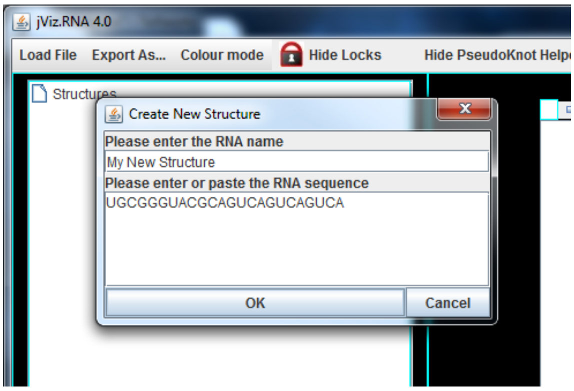

editing functionality to connect the nucleotides as you please, and the structure exporting functionality to save the structure for future work.
